# Scots Pines With Tolerance to *Melampsora pinitorqua* and *Diplodia sapinea* Show Distinct Metabolic Profiles

**DOI:** 10.1111/pce.15218

**Published:** 2024-10-25

**Authors:** Matilda Stein Åslund, Michael Reichelt, Ke Zhang, Carles Castaño, Jan Stenlid, Jonathan Gershenzon, Malin Elfstrand

**Affiliations:** ^1^ Department of Forest Mycology and Plant Pathology Swedish University of Agricultural Sciences Uppsala Sweden; ^2^ Max Planck Institute for Chemical Ecology Jena Germany

**Keywords:** chemical defence, Diplodia tip blight, disease tolerance, *Melampsora populnea*, pine twisting rust, *Pinus sylvestris*, *Sphaeropsis sapinea*

## Abstract

*Diplodia sapinea* causes Diplodia tip blight (DTB) and is recognised as an opportunistic necrotrophic pathogen affecting conifers. While DTB is associated with abiotic stress, the impact of biotic stress in the host on *D. sapinea*'s lifestyle shift is unknown. Observed co‐occurrences of *D. sapinea* and *Melampsora pinitorqua*, causing pine twisting rust on Scots pine (*Pinus sylvestris*), instigated an investigation into their interaction with and influence on the defence mechanisms of the host. We hypothesised that *M. pinitorqua* infections predispose the trees to *D. sapinea* by stressing the host and altering the shoot metabolites. Pines in a plantation were sampled over time to study pathogen biomass and host metabolites. Symptoms of both pathogens were consistent over years, and the preceding season's symptoms affected the metabolic profiles pre‐infection and *M. pinitorqua*'s proliferation. Symptoms of *M. pinitorqua* altered shoot metabolites more than fungal biomass, with co‐symptomatic trees exhibiting elevated *M. pinitorqua* biomass. Specific phenolic compounds had a strong positive association with the shoot symptom × *D. sapinea* interaction. *D. sapinea*'s biomass presymptoms was independent of previous disease symptoms and infection by *M. pinitorqua*. Some trees showed disease tolerance, with delayed rust infections and minimal DTB symptoms. Further investigations on this trait are needed.

## Summary statement

This study examines the dynamics of disease outbreaks caused by the emerging pathogen *D. sapinea* in Scots pines in northern Europe, concurrently infected by the native pathogen *M. pinitorqua*. Disease symptoms vary across the studied trees, from severely affected trees to those that show tolerance by delayed rust infections and low Diplodia tip blight disease levels. Disease symptoms persist over the years and predict the pathogen dynamics, phenolic profiles and tree vitality. *M. pinitorqua* infection does not directly impact *D. sapinea* colonisation.

## 1 Introduction

Plant diseases have a profound impact on plant metabolism and physiology (Berens et al. 2017). In natural habitats, plants often face multiple pathogens with distinct modes of action (Tollenaere, Susi, and Laine 2016). It is not uncommon for them to encounter opportunistic pathogens, which thrive during altered physiological states and stress within the host. Opportunistic fungi can reside as asymptomatic endophytes, remaining latent until specific host and environmental factors convert them into aggressive necrotrophs (Slippers and Wingfield 2007). The molecular and metabolic processes driving the vulnerability of trees to opportunistic fungi, triggered by stress, are still largely unknown. This gap in knowledge makes predicting the dynamics of these pathosystems a challenging task (Ghosh et al. 2022).

*Diplodia sapinea* (Fr.) Fuckel (syn. *Diplodia pinea* (Desm.) Kickx., *Sphaeropsis sapinea* (Fr.: Fr.) Dyko & Sutton) causes Diplodia tip blight (DTB) and has repeatedly been reported to be an opportunistic necrotrophic pathogen on conifers (Blodgett, Kruger, and Stanosz 1997; Blumenstein et al. 2021; Brodde et al. 2023; Stanosz et al. 2001; Swart, 1991; Zwolinski, Swart, and Wingfield 1995), in particularly on *Pinus* spp. (CABI 2021). *Diplodia sapinea* is a major pine pathogen globally, but the reports of damages have increased in northern Europe over the past decade (Brodde 2023; Brodde et al. 2019; Terhonen et al. 2021), with its impact expected to escalate due to climate change (Fabre et al. 2011; Sturrock et al. 2011). It is known that DTB develops when the tree is under the influence of abiotic stressors such as drought, hail, or mechanical damage (Blodgett, Kruger, and Stanosz 1997; Brodde et al. 2023; Sherwood et al. 2015; Stanosz et al. 2001; Swart, 1991; Zwolinski, Swart, and Wingfield 1995). In artificial inoculation experiments on Scots pine (*Pinus sylvestris* L.), wounding facilitated a higher incidence of symptomatic *D. sapinea* infections and significantly increased the success of pathogen reisolation, while non‐wounded plants predominantly exhibited asymptomatic infections (Oostlander et al. 2023), indicating that stress in the host may allow *D. sapinea* to transition into an aggressive necrotroph.

Both latent *Diplodia sapinea* infection and acute DTB may induce alterations in both primary and secondary metabolism of the host (Ghosh et al. 2022; Hu et al. 2023), causing local carbon and nitrogen stress in the host tissues (Ghosh et al. 2022; Sherwood et al. 2015). Infection by *D. sapinea* on pines is associated with the accumulation of lignin, phenolics and free amino acids (Hu et al. 2023; Sherwood et al. 2015; Wallis et al. 2008). The accumulation of phenolic glycosides and stilbenes has shown a negative correlation with disease susceptibility (Wallis et al. 2008). In a study on *D. sapinea* in hail‐damaged and non‐hail‐damaged pine stands, Caballol et al. (2022) proposed a potential proline competition between other endophytes and *D. sapinea*, influencing the disease outcome in pines affected by hail. Among the free amino acids, proline has been reported to be a preferred nitrogen source by *D. sapinea* (Sherwood et al. 2015). Therefore, Sherwood et al. (2015) suggest that nitrogen availability may play a pivotal role in shaping the outcome of the interaction between pine and *D. sapinea* and that the availability or increased abundance of free amino acids in stressed trees may contribute to disease development by providing *D. sapinea* with nitrogen. In their paper, Zwolinski, Swart and Wingfield (1995) suggested the potential of cambiophagous insects to infest healthy radiata pine (*P. radiata*) tissue and facilitate further colonisation by *D. sapinea*, indicating that biotic stress may also allow *D. sapinea* to change from an endophytic to a necrotrophic lifestyle.

Pathogens can alter signals that modify host defence responses and host metabolism. Reactions triggered by one pathogen can also be changed in the presence of another (Abdullah et al. 2017). Priority effects stem from the host's immune responses to earlier pathogen infections and may arise when a previous infection changes the susceptibility to subsequent infections (Halliday, Umbanhowar, and Mitchell 2018). Prioritising defence against certain pathogens increases investment in defending against them but may weaken defences against others (Abdullah et al. 2017). The process of immune‐mediated facilitation can thereby occur when one immune‐signalling pathway's upregulation leads to another's downregulation, facilitating subsequent infections and increasing co‐infection frequency (Halliday, Umbanhowar, and Mitchell 2018).

The defence mechanisms of conifers are multifaceted, functioning at different stages of infection and disease progression. The defences are constitutive or induced, chemical or mechanical, and systemic or local (Fraser et al. 2016). Conifers allocate different types of chemical defences in designated structures as they grow (Franceschi et al. 2005; Nerg et al. 1994). They also often activate multiple defences, including various phenolic compounds, as a response to pathogen attacks (Fraser et al. 2016; Villari et al. 2014). Phenolics encompass a wide array of metabolites originating from the shikimate pathway, like flavonoids, stilbenes, and lignins and their precursors. The mode of action is through direct toxic effects, inhibition of extracellular enzymes generated by pathogens, or the prompt deposition of barriers like lignin (Bennett and Wallsgrove 1994; Fraser et al. 2016; Ullah et al. 2017). It has been reported that phenolic compounds may act as a reservoir for the synthesis of other phenolic compounds when the phenylpropanoid metabolism is activated in induced defences (Keinänen et al. 1999; Lamara et al. 2018). While this may allow a faster response to environmental threats, it may also potentially influence the host's defence responses against other attackers.

Scots pine account for approximately 40% of the standing volume in Sweden (SLU 2023). One of the most prevalent diseases affecting Scots pine in the country is pine twisting rust (Skogsstyrelsen 2023), caused by the biotrophic rust fungus *Melampsora pinitorqua* (Braun) Rostrup (syn. *Melampsora populnea* (Pers.) P. Karst.). This fungus alternates between European aspen (*Populus tremula* L.) and Scots pine, thriving particularly in newly established pine plantations where aspen often emerges. *M. pinitorqua* teliospores overwinter on aspen leaves on the ground, forming basidiospores in spring that infect flushing pine shoots (Klingström 1963). The duration of infection on pine is brief, but it leaves behind a canker that often induces the shoot to bend or break, and if the leader shoot is affected, it can lead to deformed or multiple stems. Susceptibility to *M. pinitorqua* positively correlates with tree growth and vigour (Desprez‐Loustau and Wagner 1997; Desprez‐Loustau and Dupuis 1994; Klingström 1963; Martinsson 1985). Recent reports have indicated the co‐occurrence of *D. sapinea* and *M. pinitorqua* on Scots pine in Sweden, stressing the need to investigate underlying mechanisms (Skogsstyrelsen 2023).

Rust fungi depend entirely on energy and nutrients from living plant host cells to complete their lifecycle (Lorrain et al. 2019). These fungi, including *M. pinitorqua*, employ effector proteins to suppress host defence responses and extract carbon directly from living cells, creating a local carbon sink (Oliva, Stenlid, and Martínez‐Vilalta 2014). The signatures in their genome indicate that host oligopeptides are a source of essential nitrogen and sulfur for the rust fungi (Guerillot et al. 2023; Lorrain et al. 2019). Recently, it was shown that changes in the content of specific amino acids, flavonoids and terpenoids in crabapple leaves following infections with the rust fungus *Gymnosporangium yamadae* is associated with an increased abundance of specific taxonomic groups, such as *Venturiaceae*, which includes several plant pathogens, in the host mycobiome (Zhang et al. 2023). Considering our understanding of the metabolic cues that trigger the lifestyle shift in *D. sapinea* and rust fungi's impact on host metabolism and defence responses, we anticipated that the defence responses triggered by *M. pinitorqua* could potentially facilitate infection by *D. sapinea*.

In this study, we aimed to understand the interactions between Scots pine and two prevalent fungal pathogens and investigate how they influence the tree defence mechanisms. We used a site in Västmanland, central Sweden, that was established in 2015. Two years later, forest managers began reporting significant issues with *M. pinitorqua* on the otherwise vital and well‐growing pines in the area, and in 2020, at a closer inspection, it was discovered that *D. sapinea* infected many *M. pinitorqua*‐symptomatic trees. Those findings led us to hypothesise that *M. pinitorqua* infection predisposed the trees to new infections by *D. sapinea* existing in the environment, and we additionally formulated three more specific hypotheses: 1) the disease symptoms of the tree in the preceding growing season impact the amino acids and phenolics profile, pathogen biomass abundance, and the tree's vitality during the following growing season, 2) the composition of amino acids and phenolics differs between *M. pinitorqua*‐symptomatic and asymptomatic tissue, and that 3) tissues from shoots colonised by *M. pinitorqua* but not by *D. sapinea* exhibit a distinct set of phenolic compounds compared to tissues colonised by both pathogens. To test these hypotheses, we selected 15 trees based on their disease symptoms in 2020: five healthy‐looking, five with *M. pinitorqua* symptoms, and five with both *M. pinitorqua* and *D. sapinea* symptoms. We sampled symptomatic and asymptomatic shoots at three time points, comparing amino acid and phenolic profiles and the abundances of *M. pinitorqua* and *D. sapinea*.

## 2 Materials and Methods

### 2.1 Experimental Site and Plant Material

The experimental site is located outside Ängelsberg, Västmanland, Sweden (N 59.956820, E 16.059968). The site experienced an extensive forest fire in 2014 that consumed all the surface vegetation and even fractured the bedrock beneath. In 2015, the area was reforested with 1‐year‐old Scots pine seedlings, each placed in a heap of mineral soil (approximately 20 cm high). In the spring of 2021, 567 trees with heights ranging from 100 to 275 cm were surveyed for *M. pinitorqua* and *D. sapinea* symptoms. Based on the survey, five of the healthiest trees (with the lowest percentage of *M. pinitorqua*‐infected shoots and negligible or no *D. sapinea* infections, disease category H), five of the trees with the greatest number of *M. pinitorqua* infections but few or no visually discernible *D. sapinea* infections (disease category M), and five of the trees with the highest percentage of *M. pinitorqua*‐infected shoots and greatest number of infections by *D. sapinea* (disease category MD) were chosen for detailed investigations. Representative photos of trees from the three disease categories are presented in Supporting Information S1: Figure S1, and the phenotyping data is summarised in Table S1.

### 2.2 Survey of *M. pinitorqua* and *D. sapinea* Symptoms

#### 2.2.1 *Melampsora pinitorqua* Symptoms

In spring 2021, we surveyed the trees for the percentage of shoots in the top whorl of the previous year (2020) and the percentage of previous year shoots in the second whorl (15 random shoots surveyed) showing infections by *M. pinitorqua*. The percentage of shoots infected by *M. pinitorqua* in the top whorl of 2019 was also estimated.

In autumn 2021, the survey was repeated, recording the percentage of shoots in the top whorl of 2021 and the percentage of current year shoots in the second whorl (15 random shoots surveyed) infected by *M. pinitorqua*.

#### 2.2.2 *Diplodia sapinea* Symptoms

Shoot samples were collected from twelve trees in September 2020 to confirm the presence of *D. sapinea* based on the morphology of the conidia using a microscope. For all trees included in the study, the presence/absence of *D. sapinea* infections in the top shoot and the total number of visible *D. sapinea* infections (based on typical symptoms) were recorded in spring 2021 (for shoots from 2020) and in autumn 2021 (for shoots from 2021).

#### 2.2.3 Growth and Vitality

Measurements were taken for tree height at the end of the growing season in 2021, as well as height growth from mid‐node to mid‐node for the years 2018 through 2021.

In autumn 2021, the general condition of each tree was scored based on how affected their appearance and growth were due to symptoms by the two pathogens. A scale of 0–5 was used, where 0—fully vital, no signs of disease, 1—fully vital, 2—mildly affected, 3—affected, 4—clearly affected, and 5—severely affected. The trees were later grouped into three vitality classes: fully vital (0–1), mildly affected (2–3), and severely affected (4–5). Representative photos of trees from the three vitality classes are presented in Supporting Information S1: Figure S2.

### 2.3 Sampling of Scots Pine Shoot Tissue for Quantification of Metabolites, *M. pinitorqua* DNA, and *D. sapinea* DNA

**Figure 1:**
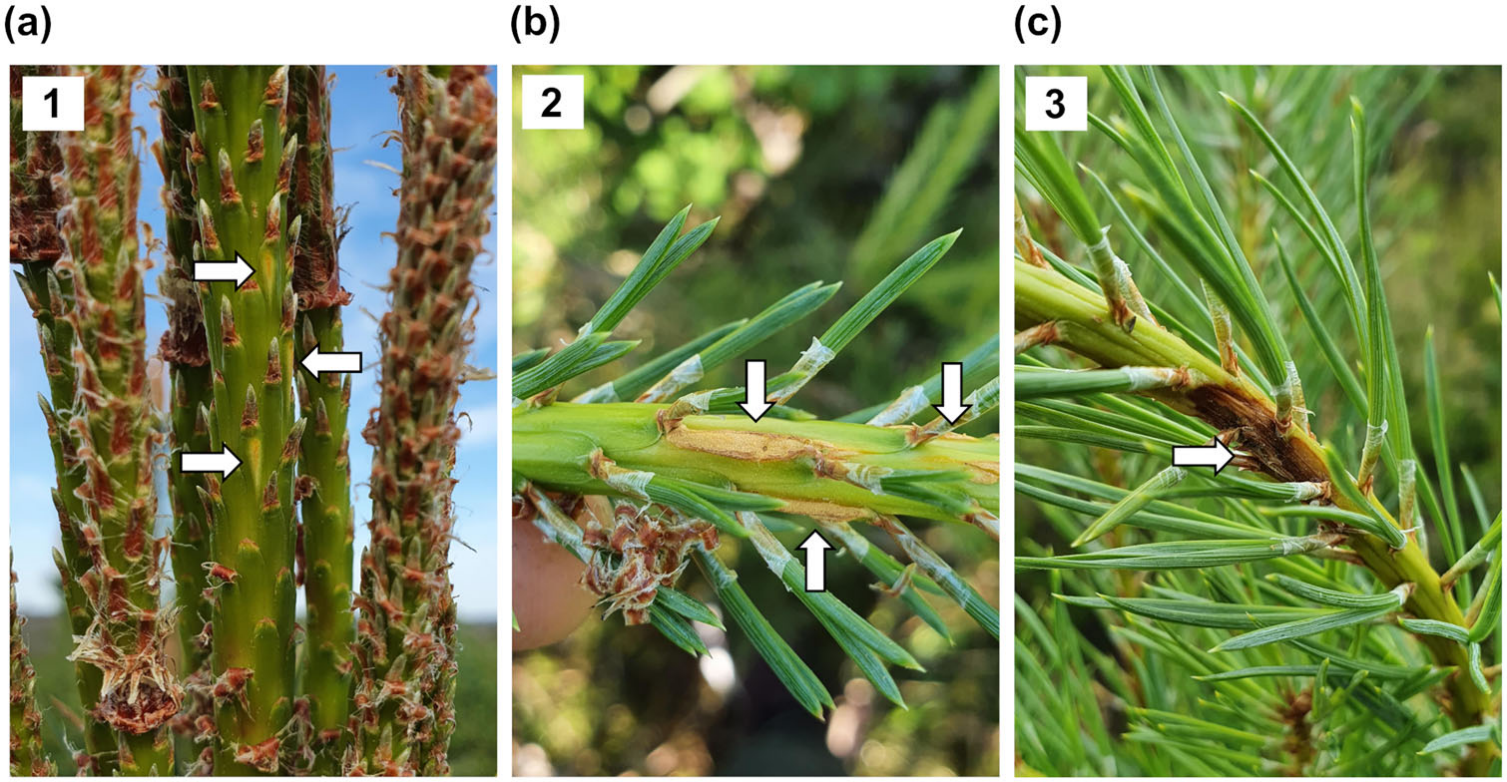
Representative photos of the *M. pinitorqua* infection phase on Scots pine shoots at the different sampling time points. White arrows point to symptoms of *M. pinitorqua*. (a) Time point 1 (2 June 2021)—early signs of aecidia on the shoots, (b) time point 2 (10 June 2021)—aeciospores on the surface of the infection site, (c) time point 3 (8 July 2021)—infection inactive and the canker has started to heal.

Sampling was done during three time points in 2021; when *M. pinitorqua* infections were first visible on the shoots (2 June), when *M. pinitorqua* aeciospores were visible (10 June), and when the trees had started healing the wounds caused by the infections (8 July). No signs of *D. sapinea* infections were visible in young shoots at the time of sampling. Representative photos of *M. pinitorqua* infection phases from the time points are presented in Figure 1. The sampling scheme is presented in Supporting Information S1: Figure S3. The phenotyping data is summarised in Table S1, and the number of samples per time point, group, and shoot symptom is presented in Table S2.

From category M and MD, *M. pinitorqua* symptomatic and asymptomatic shoots from the same branch (where possible, otherwise from the same side of the tree) on the second branch‐whorl were cut using secateurs disinfected with 70% EtOH. No symptomatic shoots were observed on category H during the first and second time points; hence, only asymptomatic samples were collected at those time points. The samples were put straight in a cooler (filled with clamps frozen at −80°C), kept below 0°C during transport, and stored at −80°C until handling. Eighty samples were included in the metabolite and fungal biomass analyses. To prepare the samples, needles were removed using scissors, and the top of each shoot was removed to fit the sample in a 15 mL Falcon tube. The shoot surface was washed by adding ~12 mL 0.01% Tween, shaking the tubes and then rinsing the samples in ddH_2_O three times. Each sample was cut using a disinfected scalpel adjacent to and above the *M. pinitorqua* infection site to a length of ~25 mm. Asymptomatic samples were cut at the corresponding height and to the same length. The samples were lyophilised and homogenised in 2 mL screw‐cap tubes containing an M6 screw in the bottom and an M6 nut on top by beating it in a Precellys (Bertin technologies) for 20 s at 4000 rpm as many times as needed (1‐8 times) until the sample was homogenised. A subsample of 10–20 mg of each sample was weighed and used for metabolite analysis.

### 2.4 Extraction and Quantification of *D. sapinea* and *M. pinitorqua* DNA

DNA was isolated from the remaining homogenised sample using the Qiagen DNeasy Plant mini kit with the following modifications to the protocol: (i) 700 µL AP1 and 225 µL P3 were added for tissue lysis; (ii) 450 µL lysate were transferred to the QIAshredder spin column; and (iii) DNA was eluted twice.

Primers for *M. pinitorqua* diagnostics and quantification were designed in‐house (F: 5′ CCC TCG GCT TTA ACA CTT TCT A‐3′, R: 5′‐CGA TAC GAC CAA AGA CCA TCT C‐3′). Briefly, the genus‐specific region was identified in an alignment of the ITS1‐5.8S‐ITS2 region of *Melampsora* spp. and other closely related rust species and used to design the primers. The primers amplify a 168‐bp fragment in the ITS2 region of *Melampsora* spp. The specificity was confirmed with (1) NCBI primer blast: among the 251 returned results, 96% were *Melampsora* spp, and the remainder sequences were uncultured fungi except one (GQ479878, labelled as nematode); (2) standard PCR with common rust fungi, Scots pine pathogens and endophytes: *Cronartium pini, Coleosporium* sp., *Gymnosporangium* sp., *M. larci‐epitea, M. pinitorqua, Puccinia triticina, P. graminis, Thekopsora areolata, Aequabiliella palatina, Cladosporium* sp., *Sarea coeloplata, and Sydowia polyspora*. Only *Melampsora* spp. produced products with the expected size. The GH3 homologue for the determination of Scots pine biomass in qPCR reactions was designed by Heller et al. (2012). For the determination of *D. sapinea* biomass, the assay used for qPCR was designed by Luchi et al. (2005).

Standard curves for qPCRs for *M. pinitorqua*, *D. sapinea*, and Scots pine were produced by PCR amplification on DNA templates from *M. pinitorqua* (aeciospore sample), *D. sapinea*, and Scots pine. The PCR products' desired lengths (*M. pinitorqua* 168 bp, *D. sapinea* 79 bp, Scots pine ~100 bp) were confirmed through gel electrophoresis. DNA from the PCR products were precipitated, quantified using a Qubit Fluorometer, and used for tenfold serial dilutions to generate standard curves for assays for each organism. The qPCR reactions for quantification of *M. pinitorqua* and Scots pine contained 1× SsoFast EvaGreen Supermix (Bio‐Rad) and 500 nM of forward and reverse primer, respectively. The qPCR reactions for quantification of *D. sapinea* followed the protocol by Luchi et al. (2005) with the following modifications: 1× SsoAdvanced™ Universal Probes Supermix (Bio‐Rad), 250 nM each of forward and reverse primer, and 200 nM probe. The reaction volumes were 15 μL, using either 12.5 ng of DNA template or ddH_2_O as a nontemplate control. All assays were conducted using the CFX Maestro qPCR detection system (BioRad) with the same cycling conditions: 2 min at 95°C and 40 cycles at 95°C for 10 s and 60°C for 15 s. Each assay included a standard curve of serial dilutions from 1 × 10^7^ to 1 × 10^2^ copies per reaction (in duplicates) and three non‐template controls. qPCR efficiencies ranged from 94.5% to 97.3% for Scots pine, 90.2%–96.9% for *M. pinitorqua* and 90.2%–96.7% for *D. sapinea*. All assays had an *R* ^2^ > 0.99. CFX Maestro software (version 5.3.022.1030) (BioRad) was used to analyse the qPCR data. Samples were excluded if the cycle threshold (Ct) value's standard deviation (stDev) among replicates was > 0.5 (occurring only for *M. pinitorqua* assays, *n* = 2). Samples below the assay's linear detection limit (37.0 Ct) were included in the analysis with SQmean = 0, even if the stDev was > 0.5.

The qPCR copy numbers for *M. pinitorqua* and *D. sapinea* were normalised based on the amount of Scots pine DNA in the same sample (the desired amount of DNA per reaction was 12.5 ng, corresponding to ~625 haploid pine genomes).

### 2.5 Extraction and Quantification of Amino Acids and Phenolic Compounds

#### 2.5.1 Phenolic Compounds

The methanol extracts (1 mL methanol per sample (10–20 mg) containing 10 μg/mL apigenin‐7‐glucoside as an internal standard) were first run on an LC‐UV‐Ion‐Trap‐MS (1100 series equipment (Agilent Technologies, Germany)) coupled to an Esquire 6000 ESI‐Ion Trap mass spectrometer (Bruker Daltonics, Germany) to find peaks that absorb at 280 or 330 nm and to determine their molecular weights. Later the samples were analysed for quantification by LC‐MS/MS. Chromatography was performed on an Agilent 1200 HPLC system (Agilent Technologies, Boeblingen, Germany). Separation was achieved on a Zorbax Eclipse XDB‐C18 column (50 × 4.6 mm, 1.8 µm, Agilent Technologies). Formic acid (0.05%) in water and acetonitrile were employed as mobile phases A and B, respectively. The elution profile was: 0.0–1.0 min 0%; 1.0–7.0 min, 0%–65% B; 7.0–7.01 min, 65%–100% B; 7.01–8.0 min 100% B, and 8.01–10.0 min 0% B. The mobile phase flow rate was 1.1 mL/min. The column temperature was maintained at 25°C.

An API 3200 tandem mass spectrometer (Applied Biosystems, Darmstadt, Germany) equipped with a Turbospray ion source was operated in negative ionisation mode. The ion spray voltage was maintained at −4200 eV. The turbo gas temperature was set at 600°C. Nebulising gas was set at 60 psi, curtain gas at 30 psi, heating gas at 60 psi, and collision gas at 6 psi. The mass spectrometer was operated in multiple reaction monitoring (MRM) mode; details of the instrument parameters can be found in Table S3. Both Q1 and Q3 quadrupoles were maintained at unit resolution. Analyst 1.5 software (Applied Biosystems, Darmstadt, Germany) was used for data acquisition and processing. Linearity in ionisation efficiencies was verified by analysing the dilution series of standard mixtures.

The following compounds were quantified absolutely from available standards and determined response factors: catechin, taxifolin, astringin, proanthocyanidin B1, isorhamnetin, piceid, quercetin‐glucoside, taxifolin‐glucoside, and kaempferol‐3‐glucoside. The other compounds were tentatively identified and were relatively quantified as normalised peak area/g dw: naringenin‐6‐C‐glucoside, kaempferol‐3‐(6”‐acetyl‐glucoside), isorhamnetin‐acetyl‐glucoside, kaempferol‐3‐(6”‐coumaroyl‐glucoside), kaempferol‐3‐(3”,6”‐di‐coumaroyl‐glucoside), 479‐316 (compound identity unknown; myricetin‐hexoside, molecular formula C_21_H_20_O_13_), gallocatechin, neolignan, and matairesinol. A majority of the compounds were previously described from pine species (Slimestad 2003).

#### 2.5.2 Amino Acids

Amino acids were quantified with an LC‐MS/MS using a C18‐column (XDB‐C18, 50 × 4.6 mm × 1.8 µm; Agilent, Santa Clara, CA, USA) after diluting the methanol raw extracts (used for phenolic compound analysis, see above) at 1:10 (v:v) with water containing 10 µg/mL of a mixture of 15 N, 13C‐labelled amino acids (Isotec, Miamisburg, OH, USA) and 5 µM of D5‐tryptophane (Cambridge Isotope Laboratories, Inc.; Andover, MA). For details on the chromatography and mass spectrometry (Agilent 1260 LC system (Agilent Technologies, Santa Clara, CA, USA) coupled with a QTRAP 6500 tandem mass spectrometer (AB Sciex, Darmstadt, Germany)), see Crocoll et al. (2016) and Table S4. The mass spectrometer was operated in positive ionisation mode in multiple reaction monitoring mode. All amino acids were quantified relative to the peak area of the corresponding labelled compound, except for asparagine (using aspartate and a response factor of 1.0).

The analysed metabolites are presented in Table S5a,b.

### 2.6 Statistical Analysis

Data processing, analyses and visualisation were done using R software (v. 4.1.2; R Core Team 2021). One of the samples showed extreme concentrations of several amino acids and was therefore removed from the amino acid data set. It did not appear to be an outlier in the data for phenolic compounds. When motivated, this outlier was removed from the analyses, e.g., when samples were analysed across metabolite categories. The effect of time point, shoot symptom and disease category on the amino acid and phenolic compound concentrations was tested and visualised using redundancy analysis (RDA) in the *vegan* package (Oksanen et al. 2022), with 79 independent observations and a response matrix of 19 dimensions for amino acids and 80 independent observations and a response matrix of 18 dimensions for the phenolic compounds. The RDAs were run with metabolites scaled proportionally to eigenvalues and conditioned on (i.e., statistically controlling for) time point, tree individual and shoot symptom to remove the effect of those variables when appropriate. Significances for groups were tested using permutational ANOVA. The relationship between total amino acids or phenolics and disease category time point 1 was assessed using Kruskal–Wallis rank sum test with Dunn's post hoc test performed with the kruskal.test and dunn.test functions from the packages *stats* and *dunn.test* (base R implemenation and Dinno [2017], respectively). The association between total amino acids or phenolics and shoot symptoms at each time point was evaluated using the Wilcoxon rank sum test (wilcox.test function in the package *stats*, base R implementation). Indicator Species Analysis (ISA), using the function multipatt from the package *indicspecies* (Cáceres and Legendre 2009), was then performed on rescaled data with 999 permutations to further investigate the metabolites associated with time point, shoot symptom, and disease category. Heat maps were created with the *pheatmap* package (Kolde 2019) on *Z*‐score normalised data using the complete clustering method. The effect of time point, shoot symptom, and disease category on the abundance of *M. pinitorqua* and *D. sapinea* DNA was tested using ANOVA (function aov in package *stats*, base R) on linear models. A transformation with the natural logarithm was performed on the DNA copy number data due to the deviation from a normal distribution with 1 added to each value (log(1 + x)). Estimated marginal means (package *emmeans* [Lenth 2023]) using multivariate t distribution adjustment were computed as post hoc tests for comparison between groups, and significance letters were assigned using the function cld in the package *multcomp* (Hothorn, Bretz, and Westfall 2008). To assess the relationship between individual metabolite concentration levels and pathogen biomass, generalised linear models (GLMs) with a Gamma distribution and log‐link function were fitted, including time point and tree individual as covariates, using the glm function (package *stats*, base R implementation). The association between tree vitality in 2021 and disease symptoms in 2020 was tested using a chi‐square test (chisq.test function, base R implementation). Figures were created using the *ggplot2* package (Wickham 2016) and the *pheatmap* package (Kolde 2019) in R Studio (R Core Team 2021).

## 3 Results

### 3.1 Impacts of Disease History and Co‐Infection on Phenolic Profile, Tree Vitality and Pathogen Dynamics

The disease categories assigned based on the trees' symptoms after the 2020 growing season remained stable throughout the study. The healthy‐looking (H) trees consistently had the lowest number of *M. pinitorqua*‐infected shoots from 2019 to 2021 and remained virtually free of DTB. The *M. pinitorqua*‐symptomatic (M) trees exhibited a high incidence of *M. pinitorqua* symptoms but had a low number of DTB‐symptomatic shoots during the same period. The *M. pinitorqua*‐ and *D. sapinea*‐symptomatic (MD) trees persistently had a high ratio of *M. pinitorqua*‐infected shoots and the highest incidence of DTB‐symptomatic shoots (Table S1).

Trees that were healthy‐looking in 2020 (disease category H) got infected by *M pinitorqua* later than trees that were *M. pinitorqua*‐symptomatic in 2020 (M) and trees that were *M. pinitorqua*‐ and *D. sapinea*‐symptomatic in 2020 (MD). The trees in disease category H had no *M. pinitorqua* symptoms on shoots of the top whorl in 2021 in early June (time points 1 and 2). In early July (time point 3), symptomatic shoots were found in all trees, although the frequency of infected shoots was lower in trees from disease category H than in other trees (Supporting Information S1: Figure S3, Tables S1, and S2).

**Figure 2:**
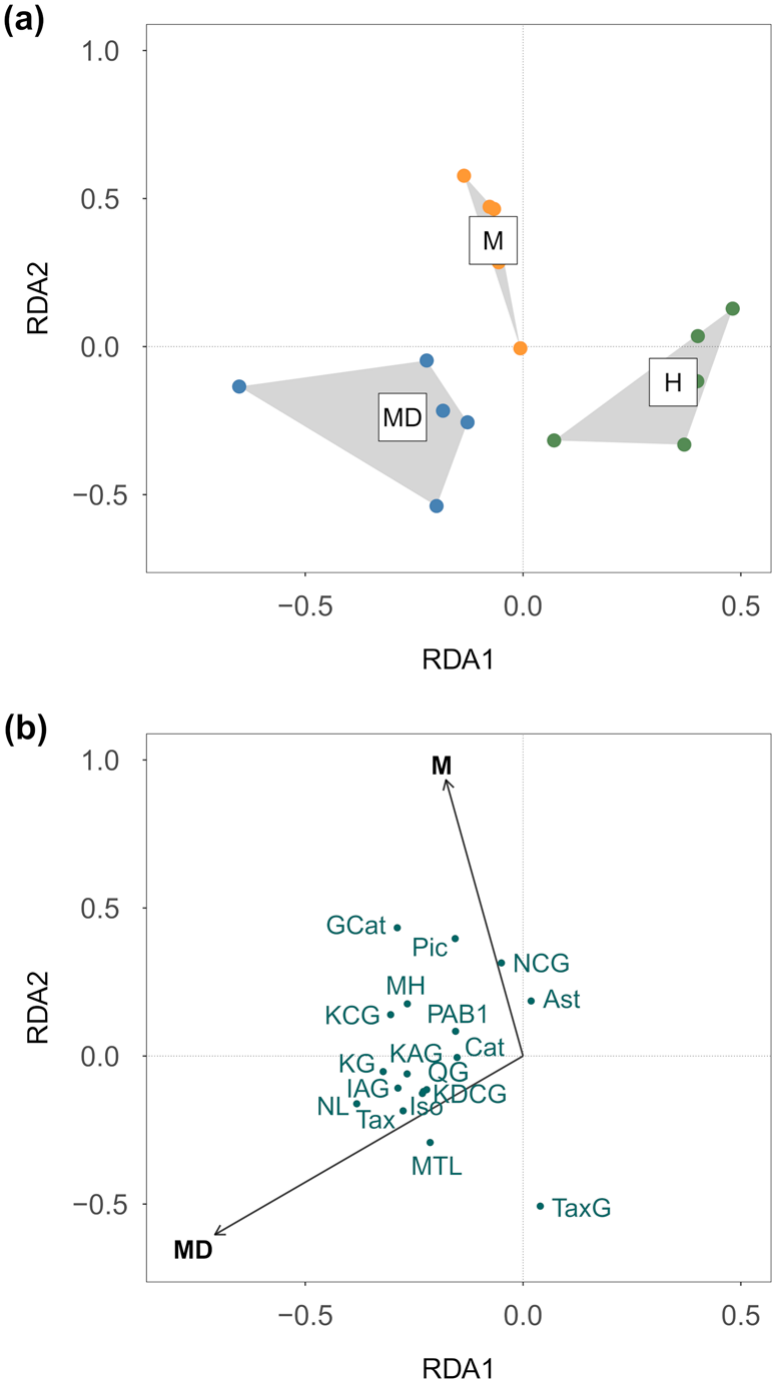
Composition of phenolic compounds in asymptomatic shoots at time point 1 (2 June 2021) explained by disease category based on the tree's symptoms in 2020; H—healthy‐looking, M—*M. pinitorqua*‐symptomatic, and MD—*M. pinitorqua*‐ and *D. sapinea*‐symptomatic. (a) Sample plot presented with hulls connecting the samples from each disease category. (b) The RDA presented with loadings. The disease category was associated with the composition of phenolic compounds (RDA; permutational ANOVA; ***p = 0.001, adj. *R* ^2^ = 0.217). For full compound names, please refer to Table 1.

For samples in the MD category, both the total amount and composition of phenolic compounds in asymptomatic shoots differed from the other two categories at the first time point (Figure 2 and Table S6a,b). The total concentration of phenolics was lower in asymptomatic shoots in the H category (Table S6b). Neither the total amount nor the amino acid profile in asymptomatic shoots at time point 1 was associated with the disease categories (Supporting Information S1: Figure S4 and Table S6a,b).

**Figure 3:**
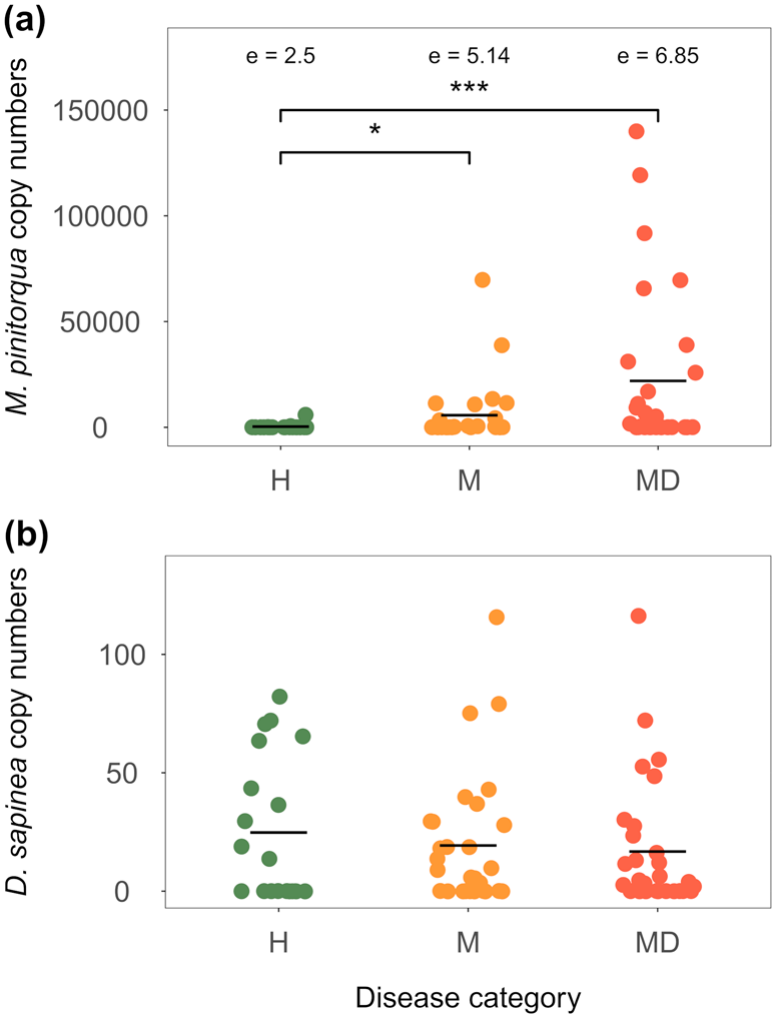
Abundance of (a) *M. pinitorqua* DNA and (b) *D. sapinea* DNA (raw copy numbers quantified using qPCR and normalised on the amount of Scots pine DNA in the sample) per disease category based on the tree's symptoms in 2020; H—healthy‐looking trees (green points), M—*M. pinitorqua*‐symptomatic trees (yellow points) and MD—*M. pinitorqua*‐ and *D. sapinea*‐symptomatic trees (red points). Black lines represent mean values, asterisks show level of significance (*p < 0.05, **p < 0.01, ***p < 0.001), ℮ shows estimated marginal means of log(1 + x).

Trees in disease category H showed lower numbers of *M. pinitorqua* DNA copies than trees in the other two categories (Figure 3a and Table S6c). There was no significant difference in the abundance of *D. sapinea* DNA between trees from different disease categories (Figure 3b, Table S6d).

The tree vitality in 2021 was significantly associated with disease symptoms in 2020 (*χ* ^2^ = 86, df = 4, ***p < 0.001). All trees categorised as healthy‐looking (H) in 2020 were in the vitality class “fully vital” in 2021. Of trees categorised as *M. pinitorqua*‐symptomatic in 2020 (M), two trees were in the vitality class “fully vital” and three trees in the vitality class “mildly affected” in 2021. One of the trees categorised as *M. pinitorqua*‐ and *D. sapinea*‐symptomatic in 2020 (MD) was in the vitality class “mildly affected” in 2021, while the four remaining trees were in the vitality class “severely affected” (Table S1).

### 3.2 Metabolic Responses to Infection by *M. pinitorqua* and *D. sapinea*

Both amino acid and phenolic compound profiles showed dissimilarities between time points (Supporting Information S1: Figure S5 and Table S6e). Thirty‐three out of the 37 metabolites contributed to the variation (Table 1). Five amino acids were identified as indicators for time point 1, two amino acids and one phenolic compound were identified for time point 2, and six phenolic compounds were identified for time point 3 (see Table 1 for details). The analysed metabolites and their concentrations are presented in Table S5a,b.

**Table 1:** Results of the indicator metabolite analyses for factors with a statistically significant multivariate effect on metabolite composition.

							Shoot symptom				Disease category (asymptomatic shoots time point 1)				
Metabolite	Abbreviation	Time point					(*M. pinitorqua*)								
		1	2	3	Stat	*p*	A	S	Stat	*p*	H	M	MD	Stat	*p*
Alanine	Ala	X			0.734	0.014									
Arginine	Arg										*No statistically significant multivariate effect of disease category was found for amino acids*.				
Asparagine	Asn	X	X		0.485	0.014									
Aspartate	Asp							X	0.302	0.016					
γ‐aminobutyric acid	GABA	X			0.602	0.014									
Glutamine	Gln	X			0.491	0.014									
Glutamate	Glu	X	X		0.412	0.025									
Histidine	His	X	X		0.584	0.014									
Isoleucine	Ile	X	X		0.601	0.014									
Leucine	Leu		X		0.328	0.014		X	0.216	0.044					
Lysine	Lys		X		0.542	0.014									
Methionine	Met	X			0.518	0.014									
Phenylalanine	Phe	X	X		0.524	0.014		X	0.219	0.033					
Proline	Pro	X			0.459	0.014									
Serine	Ser	X	X		0.703	0.014									
Threonine	Thr	X	X		0.522	0.014									
Tryptophan	Trp	X	X		0.558	0.014									
Tyrosine	Tyr	X	X		0.635	0.014									
Valine	Val	X	X		0.565	0.014									
Astringin	Ast	X		X	0.297	0.045		X	0.325	0.017					
Catechin	Cat			X	0.414	0.026		X	0.426	0.017					
Gallocatechin	GCat	X	X		0.714	0.014						X	X	0.749	0.003
Isorhamnetin	Iso			X	0.560	0.014									
Isorhamnetin‐(acetyl‐glucoside)	IAG	X	X		0.544	0.014									
Kaempferol‐3‐(3”,6”‐di‐coumaroyl‐glucoside)	KDCG		X		0.845	0.014									
Kaempferol‐3‐(6”‐acetyl‐glucoside)	KAG	X	X		0.544	0.014									
Kaempferol‐3‐(6”‐coumaroyl‐glucoside)	KCG	X	X		0.573	0.014						X	X	0.668	0.026
Kaempferol‐3‐O‐glucoside	KG	X	X		0.757	0.014	X		0.225	0.030		X	X	0.629	0.032
Matairesinol	MTL							X	0.241	0.027					
479–316^a^	MH	X	X		0.636	0.014						X	X	0.604	0.04
Neolignan	NL			X	0.656	0.014							X	0.741	0.005
Naringenin‐6‐C‐glucoside	NCG	X	X		0.466	0.039									
Proanthocyanidin B1	PAB1			X	0.495	0.026		X	0.499	0.017					
Piceid	Pic							X	0.421	0.017					
Quercetin‐glucoside	QG	X	X		0.696	0.014	X		0.202	0.042					
Taxifolin	Tax			X	0.856	0.014		X	0.178	0.031					
Taxifolin‐7‐glucoside	TaxG			X	0.857	0.014									

*Note:* Displayed are the metabolites responsible for the differences between metabolite profiles per time point (1—2 June 2021, 2—10 June 2021, 3 – 8 July 2021), *M. pinitorqua* symptom on the analysed shoot (A—asymptomatic, S—symptomatic), and disease category (based on the tree's symptoms in 2020; H—healthy‐looking, M—*M. pinitorqua*‐symptomatic, and MD—*M. pinitorqua*‐ and *D. sapinea*‐symptomatic), with their corresponding correlation statistics and permutation‐based *p*‐values (*n* permutations = 999).

^a^ Compound identity unknown; myricetin‐hexoside.

**Figure 4:**
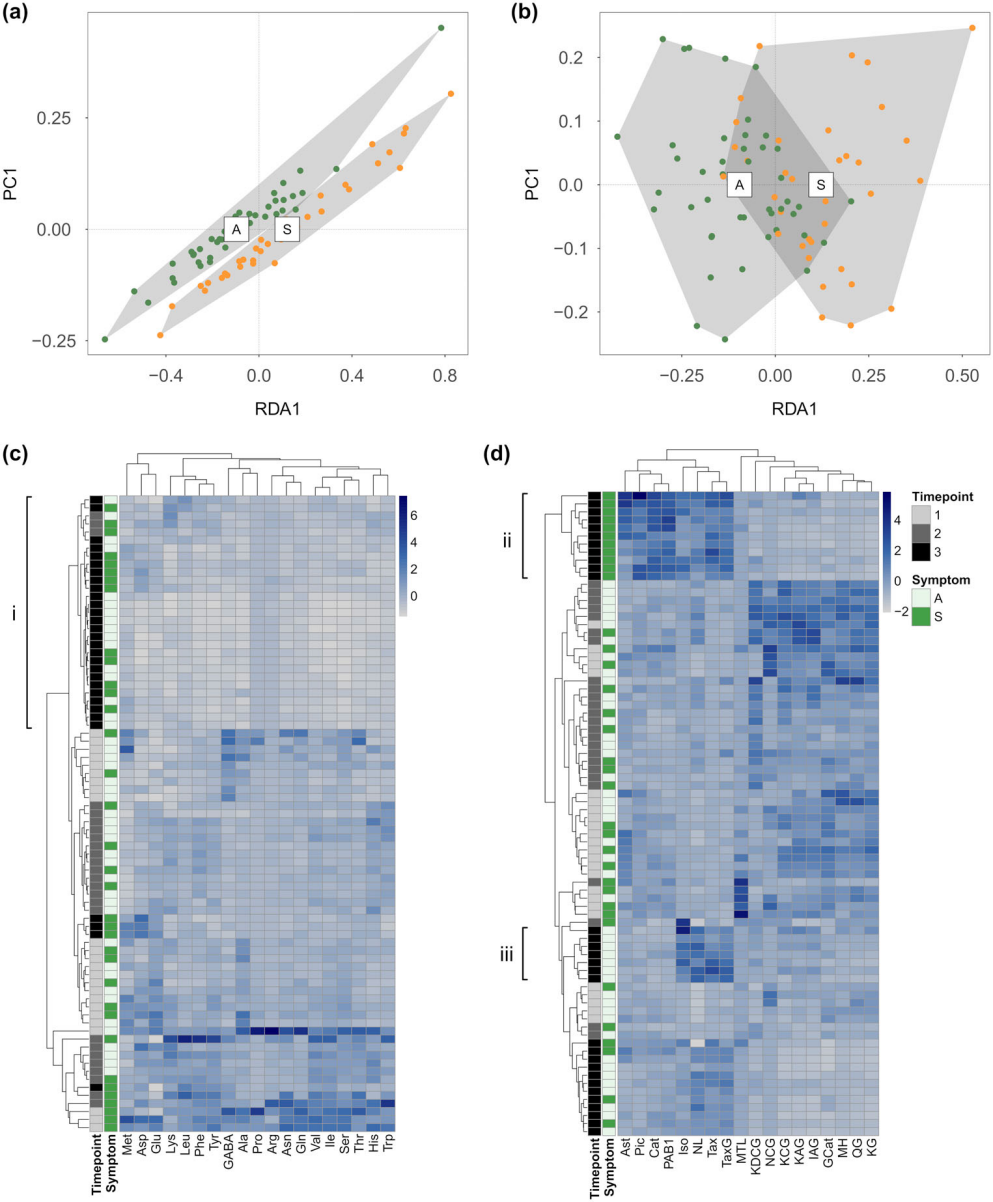
Composition of (a) amino acids and (b) phenolic compounds explained by *M. pinitorqua* symptom on the analysed shoot (A—asymptomatic, S—symptomatic). *M. pinitorqua* symptom causes significant differences in the metabolite profiles (RDA conditioned on time point and individual tree. Permutational ANOVA; *p*_amino acids_ = 0.006**, adj. *R* ^2^_amino acids_ = 0.033, *p*_phenolics_ = 0.001***, adj. *R* ^2^_phenolics_ = 0.057). Clustering of (c) amino acids and (d) phenolic compounds using Euclidean distance as the similarity measure on *Z*‐score normalised concentrations, with one pronounced cluster of amino acids in time point 3 (i) and two pronounced clusters of phenolic compounds for *M. pinitorqua*‐symptomatic shoots at time point 3 (ii) and asymptomatic shoots at time point 3 (iii). For full compound names, please refer to Table 1.

The RDA conditioned on time point showed that the amino acid profile differed between *M. pinitorqua*‐symptomatic and asymptomatic tissue (Figure 4a, Table S6f), with the strongest indicators for symptomatic shoots being aspartate, leucine and phenylalanine (Table 1). The differences between asymptomatic and symptomatic shoots were more pronounced for the phenolic compounds than for the amino acids (Figure 4b, Table S6g). The phenolic compounds that contributed most to the differentiation were proanthocyanidin B1 (PAB1), catechin, piceid, astringin, matairesinol and taxifolin, all identified as indicator metabolites for symptomatic shoots. Kaempferol‐3‐O‐glucoside and quercetin‐glucoside were indicators for asymptomatic shoots (Table 1). Both total amino acids and phenolics were higher in *M. pinitorqua*‐symptomatic shoots than in asymptomatic shoots at time point 3 (***p < 0.001, Table S6h). There was no association between shoot symptom and total amino acids or phenolics at earlier time points (Table S6h).

**Figure 5:**
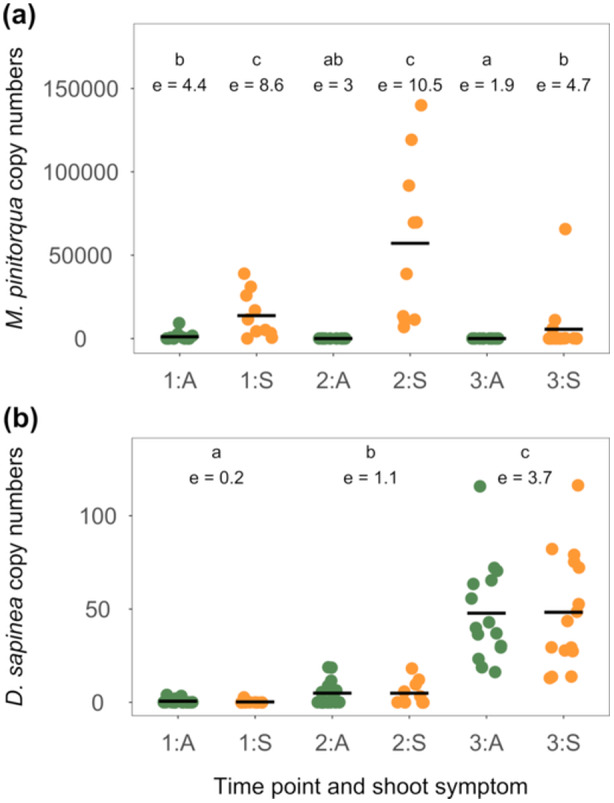
Abundance of (a) *M. pinitorqua* DNA and (b) *D. sapinea* DNA (raw copy numbers quantified using qPCR and normalised on the amount of Scots pine DNA in the sample) per time point (1—2 June 2021, 2—10 June 2021, 3—8 July 2021), and *M. pinitorqua* symptom on the analysed shoot; A—asymptomatic (green points), S—symptomatic (yellow points). Black lines represent mean values, different letters above panels indicate significant differences between groups (Tukey HSD; *p* < 0.05), ℮ shows estimated marginal means of log (1 + x).

The biomass of *M. pinitorqua*, measured as qPCR copy numbers of ITS, was higher in *M. pinitorqua*‐symptomatic shoots than in asymptomatic shoots and differed between time points; the number of DNA copies was lower at time point 3 than at both time point 1 and time point 2 (Figure 5a and Table S6c).

There was no significant deviation in *D. sapinea* biomass between *M. pinitorqua*‐symptomatic and asymptomatic shoots (Figure 5b and Table S6d). However, although no fruiting structures of *D. sapinea* were observed on the current‐year shoots, *D. sapinea* biomass increased significantly with each subsequent time point (Figure 5b and Table S6d).

**Figure 6:**
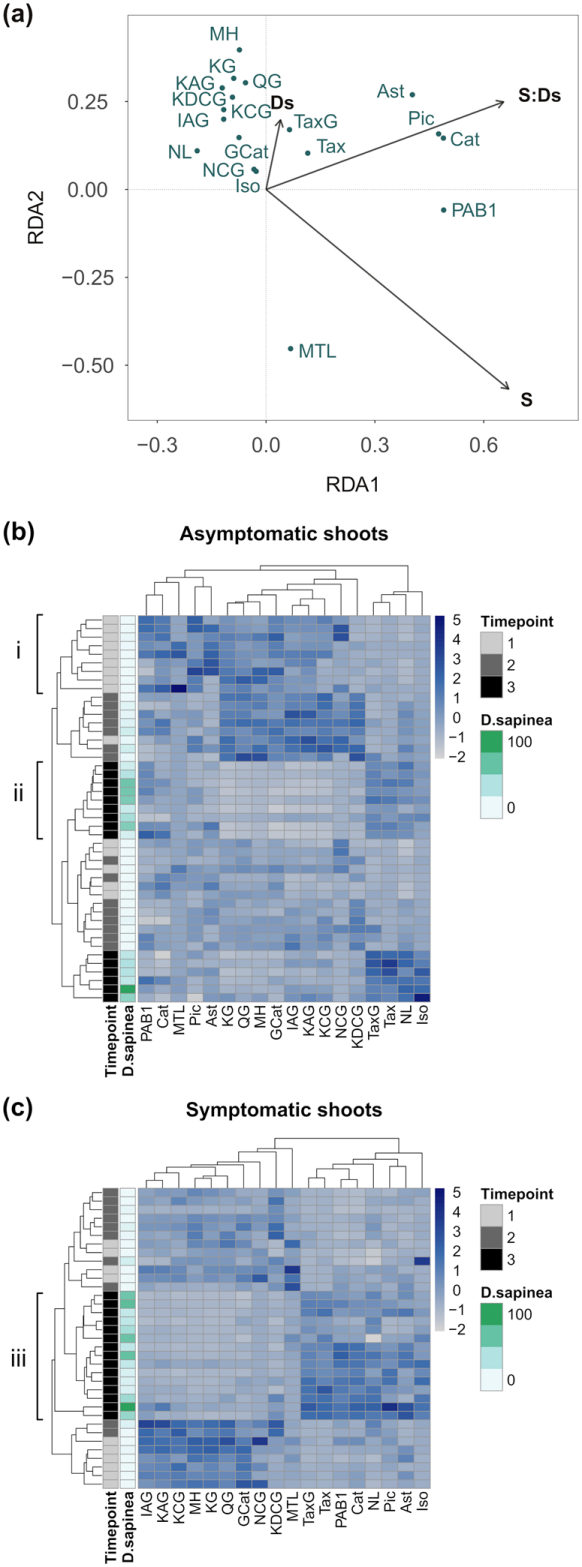
(a) Composition of phenolic compounds explained by the interaction between *M. pinitorqua* symptom (S) and abundance of *D. sapinea* DNA (Ds). Both *M. pinitorqua* symptom and the interaction with *D. sapinea* abundance influenced the phenolic compounds (RDA conditioned on time point and individual tree. Permutational ANOVA; *p_S_* = 0.001 ***, p_S×*Ds*_ = 0.001 ***, adj. *R* ^2^ = 0.106). (b and c) Clustering of phenolic compounds in (b) asymptomatic shoots and (c) *M. pinitorqua*‐symptomatic shoots using Euclidean distance as the similarity measure on *Z*‐score normalised concentrations. For full compound names, please refer to Table 1.

Neither the abundance of *M. pinitorqua* and *D. sapinea* DNA nor the interaction between their abundances was linked to the composition of amino acids or the concentration of individual amino acids or phenolics. The biomass of the individual pathogens did not relate to the phenolic compound profile, although the interaction between the pathogens was connected to the composition of phenolics. This effect was more pronounced for the interaction between *M. pinitorqua* symptom and the abundance of *D. sapinea* DNA; both shoot symptom and the interaction influenced the phenolic compounds (Table S6i). The phenolic compounds with the strongest positive association with the shoot symptom × *D. sapinea* interaction were PAB1, catechin, piceid, and astringin. Matairesinol was the metabolite with the strongest association with symptomatic shoots (Figure 6).

## 4 Discussion

In this study, we showed that the disease symptom severity caused by *M. pinitorqua* and *D. sapinea* in Scots pine trees was consistent between years. In addition, the symptoms of the preceding season affected the metabolite profiles at the beginning of the season and were associated with *M. pinitorqua*'s proliferation in the tree. *D. sapinea*'s biomass during the sampling period was independent of previous disease symptoms and infection by *M. pinitorqua*. The composition of amino acids and phenolics differed between *M. pinitorqua*‐symptomatic and asymptomatic tissue, and tissues from shoots colonised by *M. pinitorqua* but not by *D. sapinea* exhibited a distinct set of phenolic compounds compared to tissues colonised by both pathogens. However, the *M. pinitorqua* symptom had a stronger effect on the shoot metabolites than the *M. pinitorqua* biomass.

### 4.1 The Disease Categories Predict the Pathogen Dynamics, Phenolic Profiles and Tree Vitality

In 2021, trees that were healthy‐looking in 2020 (disease category H) got infected by *M. pinitorqua* later than trees that were classified as *M. pinitorqua*‐symptomatic in 2020 (M) and trees that were *M. pinitorqua*‐ and *D. sapinea*‐symptomatic in 2020 (MD). Factors such as the length of the unprotected shoot or trees falling below the height threshold for susceptibility to *M. pinitorqua* have been proposed as contributing to variation in *M. pinitorqua* infections (Desprez‐Loustau and Wagner 1997). However, there was no difference in tree height in 2021 or height growth in the years 2018 through 2021 between trees from different disease categories (data not shown). Consequently, the lower number of infected shoots on the healthy‐looking trees could not be explained by a difference in the amount of *M. pinitorqua*‐susceptible tissue between categories. The trees in disease category H also showed lower *M. pinitorqua* DNA copy numbers than trees in categories M and MD, further indicating a higher resistance in those trees and supporting our hypothesis that the abundance of pathogen biomass is impacted by the symptom status of the tree the previous year. Therefore, the population showed a quantitative variation in colonisation and symptoms, with the healthy‐looking trees (disease category H) displaying no or very few symptoms. It is possible that this observed variation is due to beneficial effects of defeated resistance (R) genes (Dowkiw and Bastien 2006). Defeated R genes refer to resistance genes previously overcome by pathogens. These genes may still confer a residual level of protection against pathogen attacks, allowing the trees that carry them to better control the disease. Furthermore, although there was no difference in *D. sapinea* biomass between trees from different disease categories, H trees showed consistently low numbers of *D. sapinea* symptoms. The H trees were the most vital trees at the end of the study. Thereby, none of the fungi were particularly successful in the H trees. These observations collectively demonstrate a higher level of disease resistance in the trees classified as healthy‐looking.

In contrast, the MD trees, which displayed symptoms of both diseases, were also the most affected at the end of the study, backing our hypothesis that the disease symptoms of the tree in the preceding growing season impact the tree's vitality during the following growing season. There are different potential explanations for this. Higher levels of *M. pinitorqua* biomass in combination with *D. sapinea* colonisation in MD trees may lead to exacerbated disease symptoms as a consequence of alterations in microbiome composition, production of toxins, or suppression of the host's immune response (Liu et al. 2023), ultimately resulting in DTB symptoms. Alternatively, MD trees may be inherently more susceptible to DTB than M trees, even when exposed to similar levels of *D. sapinea*, either due to genetic factors or physiological differences that make MD trees more vulnerable to the disease. This possibility is reflected in the observation that trees in different disease categories had different levels of total phenolics in asymptomatic tissues at time point one. This could be a genetic effect or a consequence of the disease levels in the previous season, as pathogen challenges may induce systemic accumulation of phenolics (Fossdal et al. 2012; Wallis et al. 2008). The presence of both pathogens may alter the microenvironment within the tree, creating conditions that are more favourable for DTB development. For example, the higher pathogen pressure of *M. pinitorqua* in MD trees may weaken or drain their defences (Zaman et al. 2023), making them more sensitive to *D. sapinea*, allowing the fungus to change its physiology.

### 4.2 Co‐Infections Alter the Metabolite Composition in the Shoots, But *M. pinitorqua* Infection Does Not Directly Impact *D. sapinea* Colonisation

The profiles of free amino acids in Scots pine tissues change over the season and in response to changes in the tree's environment, such as fertilisation, drought, infections, or mechanical wounding (Caballol et al. 2022; Gezelius 1986; Nasholm and Ericsson 1990; Pietiläinen and Lähdesmäki 1986). Similarly, the phenolic compound profiles change during shoot development when trees undergo rapid physiological changes to support new growth, including the synthesis of essential metabolites involved in cell division, elongation and lignification (Ghimire et al. 2019; Nerg et al. 1994). Although the first two sampling times occurred just 1 week apart, in early June, they showed distinct differences in metabolite composition. These differences are likely attributed to changes occurring during shoot development as well as during the progression of *M. pinitorqua* infections. This assumption is supported by the observed variations in both amino acid and phenolic compound compositions between tissues showing symptoms of *M. pinitorqua* and those that are asymptomatic, a finding that aligns with our hypothesis that symptoms induced by *M. pinitorqua* influence the metabolite composition within the tissue. Particularly, our analyses identified the flavonoid glucosides kaempferol‐3‐glucoside and quercetin‐glucoside as indicators for asymptomatic tissue. Flavonoids and flavonoid glucosides are normally abundant in Scots pine tissues (Laracine‐Pittet and Lebreton 1988). The flavonoid glucosides can be metabolised to aglycones, for example, quercetin‐glucoside may serve as a reservoir for quercetin or other metabolites, which can then be rapidly deployed upon pathogen attack (Keinänen et al. 1999; Lamara et al. 2018). Under such a scenario, the concentration of these compounds may decrease when symptoms appear and the metabolites are used.

The phenolic compounds contributing to the difference in the profiles were mostly indicators for symptomatic tissue, showing that the *M. pinitorqua* infection in the shoot is associated with a local accumulation of phenolic compounds. The indicator phenolics for *M. pinitorqua*‐symptomatic tissue belonged to several classes of phenolics: stilbenes (piceid, astringin), lignans (matairesinol), and flavonoids (taxifolin, catechin, PAB1). These metabolites and metabolite classes are well known to associate with defence responses in Pinaceae (Brignolas et al. 1995; Brignolas et al. 1995; Ganthaler et al. 2017; Hammerbacher et al. 2019; Harju, Venäläinen, Laakso, & Saranpää, 2009) and can be directly fungicidal or reinforce the plant cell wall, making it difficult for the pathogen to spread in the tissue (Nagy et al. 2022; Ullah et al. 2017). In short, *M. pinitorqua* symptoms locally altered metabolite composition in shoots, primarily increasing metabolite concentrations in symptomatic ones. However, the *M. pinitorqua* biomass quantity did not influence metabolite profile compositions, indicating that the responses to an active or recent *M. pinitorqua* infection may derive from the simple recognition of the infection rather than the amount of the pathogen present.

The colonisation pattern of the two pathogens differed across the sampling points. More *M. pinitorqua* biomass was consistently found in shoots with symptoms of ongoing infections at the two first sampling times, while the largest amount of *D. sapinea* biomass was detected at the third sampling point. Our sampling relied on visual assessments of disease symptoms in situ to identify and categorise *M. pinitorqua* infections. There was a good agreement between these visual assessments and the determination of *M. pinitorqua* biomass with qPCR‐based measurement of the fungal biomass; the *M. pinitorqua* biomass was generally low in asymptomatic shoots. Furthermore, the *M. pinitorqua* biomass was lowest at time point three, independent of whether the shoots were *M. pinitorqua*‐symptomatic or not. This suggests that the local infections by *M. pinitorqua* terminated after the production of aeciospores (Mattila 2005), which generally occurred between the second and third time points.

The biomass of *D. sapinea* was the highest at the third time point. Moreover, the finding that *M. pinitorqua*‐symptomatic and asymptomatic shoots contained similar levels of *D. sapinea* DNA shows that the lesions caused by the rust infections are unlikely specific entry points for *D. sapinea*, despite the frequent reports on DTB symptoms in mechanically wounded trees (Caballol et al. 2022; Oostlander et al. 2023; Smith, Wingfied, and Coutinho 2002; Zwolinski, Swart, and Wingfield 1995). The local stress responses related to *M. pinitorqua* infection, or the following healing process, also do not appear to influence *D. sapinea*'s ability to colonise the shoots. Consequently, we found no evidence that *M. pinitorqua* infections directly predisposed the trees to *D. sapinea* infection. Instead, these results support that, once *D. sapinea* is established as an endophyte, tree physiology is altered under certain conditions in a way that influences resistance to other pathogens. (Blodgett, Kruger, and Stanosz 1997; Blumenstein et al. 2021; Brodde et al. 2023; Stanosz et al. 2001; Swart, 1991; Zwolinski, Swart, and Wingfield 1995).

We expected tissues from shoots colonised by *M. pinitorqua* but not by *D. sapinea* to show a distinct set of phenolic compounds compared to tissues colonised by both pathogens, a hypothesis that proved to be true. However, the effect on the phenolic compound profile of *M. pinitorqua* symptoms was stronger than the presence of the fungus itself. The presence of *D. sapinea* in shoots with *M. pinitorqua* symptoms impacted the metabolite profiles significantly. Despite proline's and glutamate's reported importance for *D. sapinea* and the tree's defence responses (Caballol et al. 2022; Ghosh et al. 2022; Sherwood et al. 2015), we did not see any distinct patterns in the concentrations of those amino acids. The colonisation by *D. sapinea* in *M. pinitorqua*‐symptomatic tissue influenced the phenolic profiles, resulting in different concentrations of phenolic compounds compared to symptomatic tissue without *D. sapinea*. This indicates that although *M. pinitorqua* infections do not directly predispose the trees to *D. sapinea* infection, there is a local interaction between the infections shaping the profiles of phenolic compounds in the tissue when *D. sapinea* is present.

It should be emphasised that the *D. sapinea* infections appeared latent or presymptomatic when the study was carried out, and characteristic DTB symptoms were present in particular on trees in the MD category only during surveys in autumn. It is possible that the distinct changes in amino acids and phenolic compounds reported in the literature (Caballol et al. 2022; Ghosh et al. 2022; Sherwood et al. 2015) are more intimately tied to the shift in *D. sapinea*'s physiology than the direct response of the host tree to active infections. However, latent or presymptomatic infections have also been shown to induce host defence responses (e.g., Vornam et al. 2019; Hu et al. 2023). These responses can involve the expression of genes related to oxidative stress defence, lignification and flavonoid synthesis (Vornam et al. 2019) and repression of photosynthesis Hu et al. (2023).

## 5 Conclusion

Our study found no direct support for our hypothesis that *M. pinitorqua* infections predispose the trees to DTB by stressing the host and altering the composition of metabolites in infected tissues. The presence of *M. pinitorqua* symptoms on the shoot was a stronger predictor for changes in metabolite profiles than the fungal biomass. Due to the biotrophic nature of the pathogen, its recognition may be more important for the activation of the tree's defence mechanisms than the biomass. *D. sapinea* colonisation was independent of the *M. pinitorqua* biomass in the tissues. However, we also found that trees with high vitality retained vital characteristics throughout the surveyed time period and appeared to possess tolerance to *M. pinitorqua*, with delayed rust infections and minimal DTB symptoms, suggesting a more complex relationship between the host and the fungi. This potential tolerance is an interesting observation that should be followed up on in future studies, as it may be a trait that improves the resilience of young pine plantations.

## Conflicts of Interest

The authors declare no conflicts of interest.

## Supporting information

Supporting information.

## Data Availability

The data that support the findings of this study are available from the corresponding author upon reasonable request.
