# Supplementary material for: Scots Pines With Tolerance to Melampsora pinitorqua and Diplodia sapinea Show Distinct Metabolic Profiles
Source: Plant Cell Environ. 2024 Oct 25;48(2):1479–93. doi: 10.1111/pce.15218 (PMC11695801; doi:10.1111/pce.15218)
Supplement: Supplementary file 1 — Supporting information. [file PCE-48-1479-s001.docx]

**
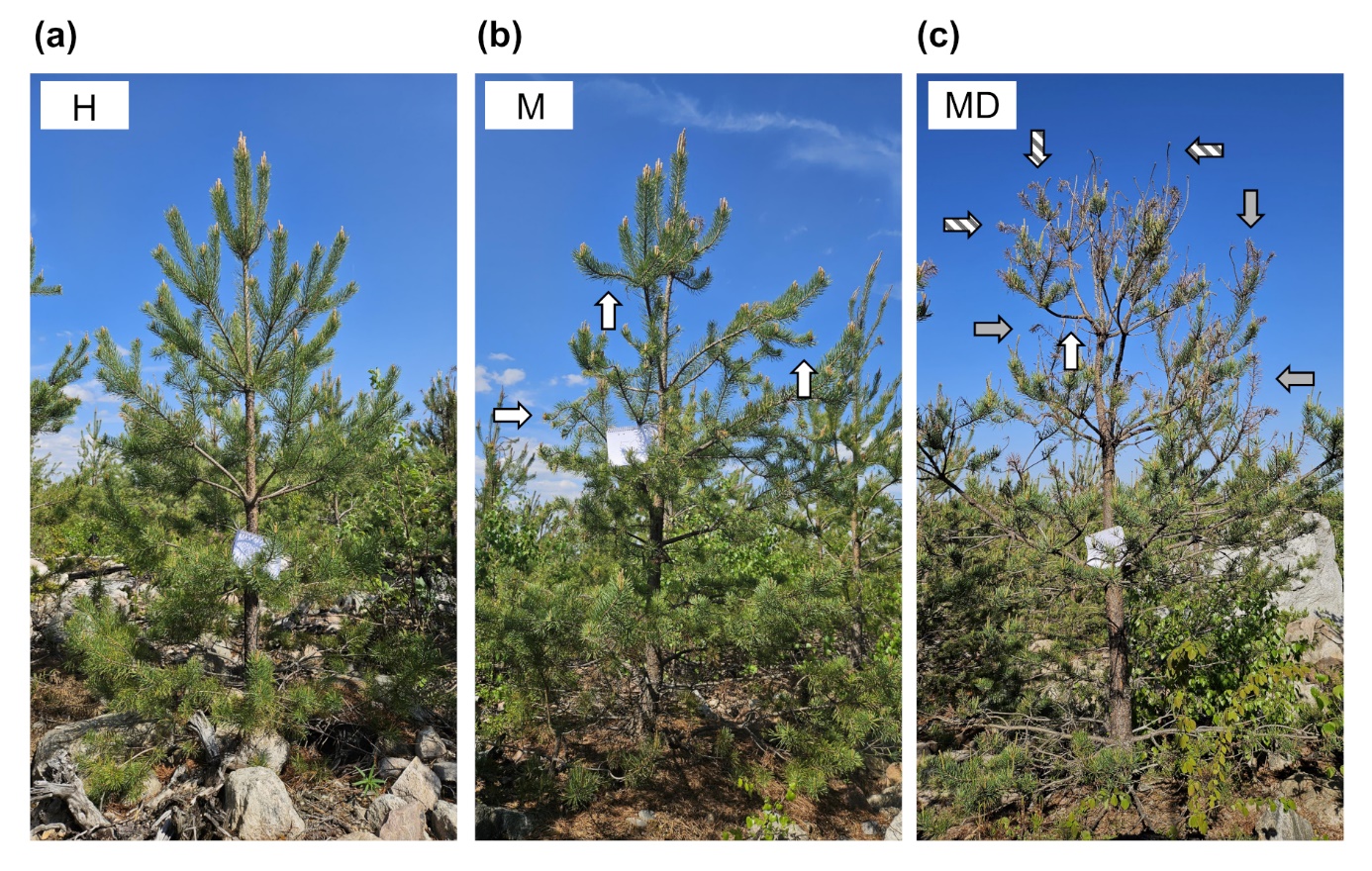

FIGURE S1** Representative photos of trees of different disease categories based on the tree’s symptoms after the growing season year 2020. White arrows point to clear symptoms of *M. pinitorqua*, grey arrows point to clear symptoms of *D. sapinea*, and striped arrows point to symptoms of both pathogens. a) Category H – healthy-looking, with few or mild *M. pinitorqua* symptoms and no signs of Diplodia tip blight; b) category M – *M. pinitorqua*-symptomatic, with bent shoots indicating previous *M. pinitorqua* infections and no signs of Diplodia tip blight; c) category MD – *M. pinitorqua*- and *D. sapinea*-symptomatic, with disease symptoms caused by both *M. pinitorqua* (bent and broken shoots) and *D. sapinea* (stunted shoot growth and dead shoots).

**TABLE S1** Tree height, yearly height growth, symptoms of *M. pinitorqua*, and symptoms of *D. sapinea* for all sampled trees (n = 15), and per disease category (based on the tree’s symptoms in 2020; H – healthy-looking, M – *M. pinitorqua*-symptomatic, and MD – *M. pinitorqua*- and *D. sapinea*-symptomatic) and vitality class in 2021. Presented are mean values (± standard error).

|  |  | **All trees** | | | | **Disease category** | | | | | | | | **Vitality** | | | | | | | |
| --- | --- | --- | --- | --- | --- | --- | --- | --- | --- | --- | --- | --- | --- | --- | --- | --- | --- | --- | --- | --- | --- |
|  |  |  |  |  |  | **H** | | |  | **M** | | **MD** | | **Fully vital** | | | | **Mildly affected** | | **Severely affected** | |
|  |  |  |  |  |  |  |  |  |  |  |  |  |  |  |  |  |  |  |  |  |  |
|  |  | n = 15 | | |  | n = 5 | | |  | n = 5 | | n = 5 | | n = 7 | | |  | n = 4 | | n = 4 | |
|  |  | **Mean** |  | **±SE** |  | **Mean** |  | **±SE** |  | **Mean** | **±SE** | **Mean** | **±SE** | **Mean** |  | **±SE** |  | **Mean** | **±SE** | **Mean** | **±SE** |
| **Tree height [cm]** | |  |  |  |  |  |  |  |  |  |  |  |  |  |  |  |  |  |  |  |  |
|  | **2021** | 215 |  | 33 |  | 234 |  | 34 |  | 218 | 20 | 194 | 34 | 229 |  | 31 |  | 225 | 22 | 182 | 23 |
| **Height growth [cm]** | |  |  |  |  |  |  |  |  |  |  |  |  |  |  |  |  |  |  |  |  |
|  | **2021** | 62 |  | 9 |  | 66 |  | 8 |  | 64 | 5 | 55 | 11 | 65 |  | 7 |  | 67 | 5 | 52 | 10 |
|  | **2020** | 41 |  | 15 |  | 53 |  | 5 |  | 39 | 16 | 32 | 14 | 50 |  | 7 |  | 40 | 19 | 27 | 9 |
|  | **2019** | 39 |  | 10 |  | 45 |  | 13 |  | 37 | 6 | 35 | 6 | 43 |  | 12 |  | 36 | 7 | 36 | 6 |
|  | **2018** | 31 |  | 6 |  | 33 |  | 8 |  | 29 | 2 | 31 | 6 | 32 |  | 7 |  | 32 | 6 | 29 | 4 |
| ***M. pinitorqua* infections (%)** | | |  |  |  |  |  |  |  |  |  |  |  |  |  |  |  |  |  |  |  |
| **1^st^ whorl** | **2021 ^a^** | 94.4 | ^d^ | 17.8 | ^d^ | 80.6 | ^d^ | 31.9 | ^d^ | 100.0 | 0.0 | 100.0 | 0.0 | 87.0 | ^d^ | 26.7 | ^d^ | 100.0 | 0.0 | 100.0 | 0.0 |
| **2^nd^ whorl** | **2021 ^b^** | 76.4 | ^d^ | 19.7 | ^d^ | 55.8 | ^d^ | 21.0 | ^d^ | 81.3 | 15.2 | 88.0 | 8.7 | 66.1 | ^d^ | 24.3 | ^d^ | 83.3 | 16.8 | 85.0 | 6.4 |
| **1^st^ whorl** | **2020 ^a^** | 81.4 |  | 28.9 |  | 50.3 |  | 31.2 |  | 95.0 | 11.2 | 98.8 | 2.8 | 60.9 |  | 32.1 |  | 100.0 | 0.0 | 98.4 | 3.1 |
| **2^nd^ whorl** | **2020 ^b^** | 59.8 |  | 37.3 |  | 15.3 |  | 21.0 |  | 78.7 | 23.8 | 85.3 | 11.0 | 33.8 |  | 36.1 |  | 80.0 | 27.2 | 85.0 | 12.6 |
| **1^st^ whorl** | **2019 ^a^** | 70.7 |  | 28.3 |  | 45.0 |  | 29.8 |  | 77.8 | 16.1 | 89.2 | 18.6 | 58.9 |  | 34.2 |  | 75.4 | 17.5 | 86.5 | 20.3 |
| **DTB-symptomatic shoots (n)** | | |  |  |  |  |  |  |  |  |  |  |  |  |  |  |  |  |  |  |  |
|  | **2021 ^c^** | 5 |  | 9 |  | 1 |  | 2 |  | 1 | 1 | 15 | 12 | 1 |  | 2 |  | 1 | 1 | 18 | 12 |
|  | **2020 ^c^** | 15 |  | 18 |  | 1 |  | 1 |  | 5 | 3 | 38 | 11 | 2 |  | 3 |  | 10 | 10 | 42 | 9 |

^a^ % of shoots with *M. pinitorqua* symptoms in the upper whorl, including leader shoot

^b^ % of shoots with *M. pinitorqua* symptoms in the ^2^nd whorl of 15 randomly selected shoots

^c^ Number of Diplodia tip blight-symptomatic shoots in crown

^d^ Value missing for one tree (n - 1)

**
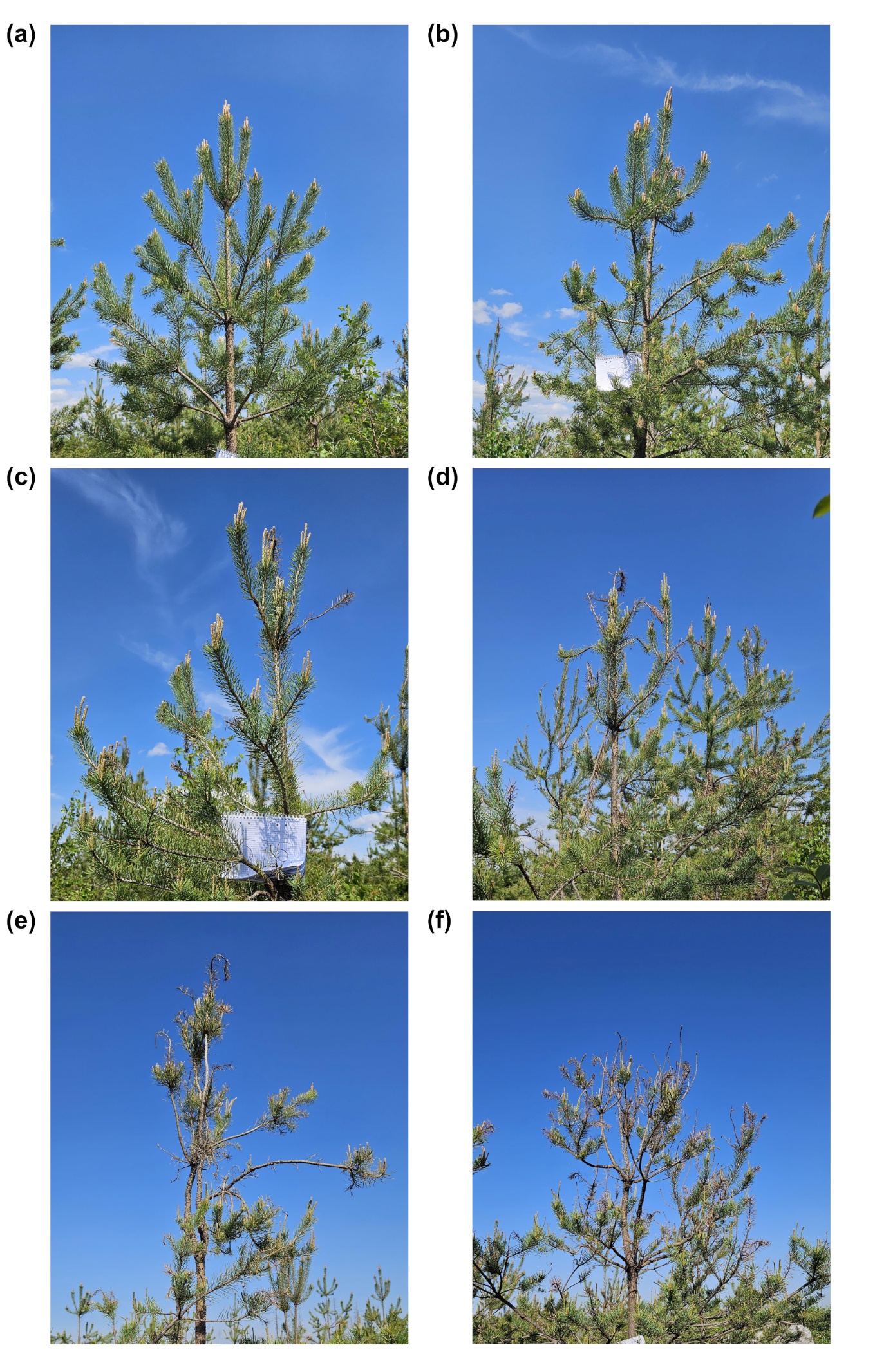

FIGURE S2** Representative photos of trees of different vitality classes Fully vital (a + b), mildly affected (c + d), and severely affected (e + f). a) No visible signs of disease. b) Bent shoots due to infection by *M. pinitorqua*, and side shoot showing symptoms of *D. sapinea* but without apparent impact on tree vitality. c) Bent shoots and moderately sparse crown due to infection by *M. pinitorqua*, scant shoots showing symptoms of *D. sapinea*, but the tree still appears vital. d) Most shoots are bent, and some are broken due to infection by *M. pinitorqua*. Several shoots show symptoms of *D. sapinea*, but the tree is not at risk of dying. e + f) Sparse crowns, double tops, many of the current year shoots are dead, clear symptoms of *M. pinitorqua* and *D. sapinea*, and risk of tree mortality.

**
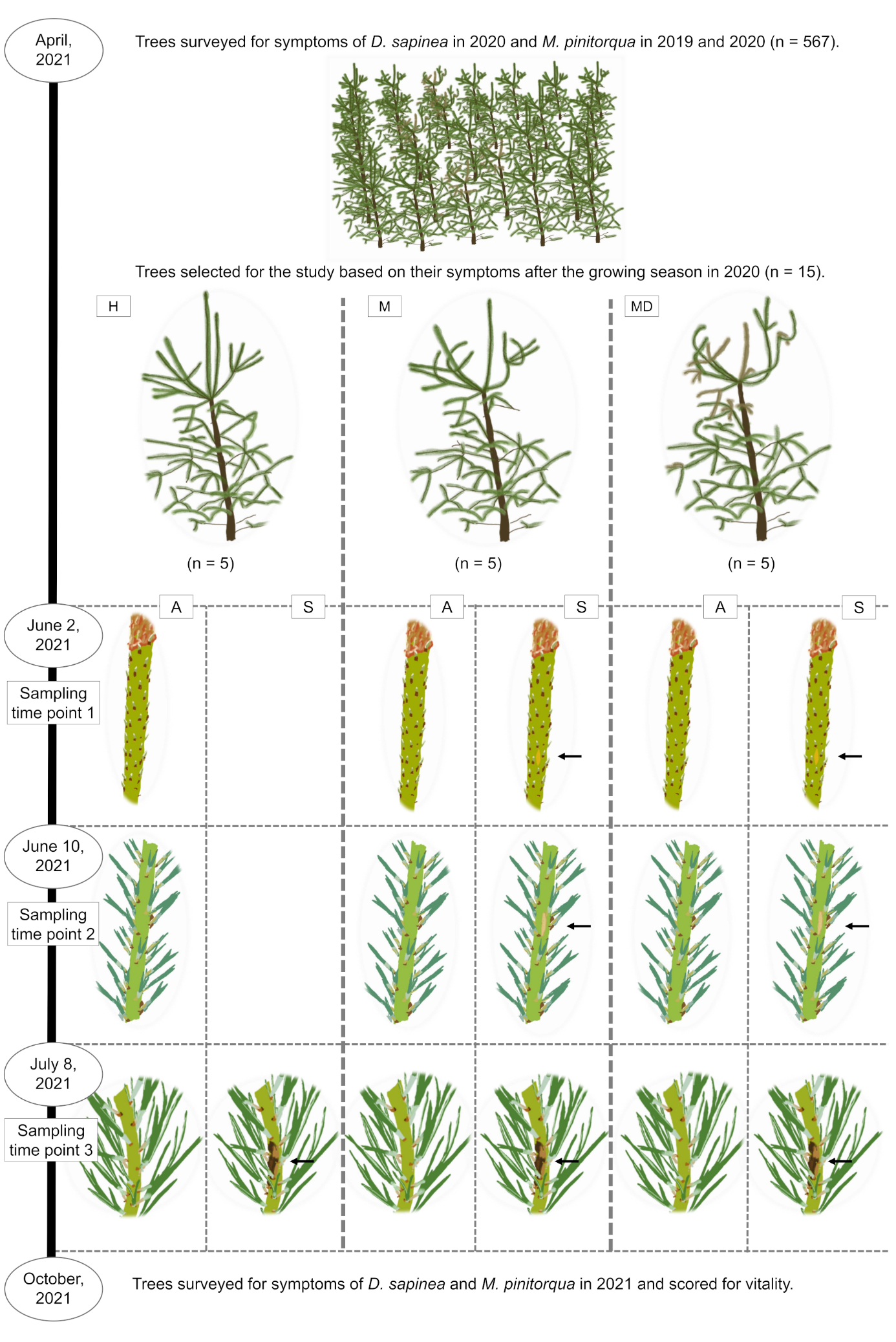

FIGURE S3** Schematic outline of the experimental design and sampling scheme. The disease categories: H – healthy-looking trees, M – *M. pinitorqua*-symptomatic trees, and MD – *M. pinitorqua*- and *D. sapinea*-symptomatic trees), *M. pinitorqua* symptom on the analysed shoot: A – asymptomatic, S – symptomatic, and time point: 1 – June 2, 2021, 2 – June 10, 2021, 3 – July 8, 2021. For exact number of samples taken for a given disease category, *M. pinitorqua* symptoms, and timepoint, please refer to Supplementary Table S1.

**TABLE S2** Samples per disease category (based on the tree’s symptoms in 2020; H – healthy-looking trees, M – *M. pinitorqua*-symptomatic trees, and MD – *M. pinitorqua*- and *D. sapinea*-symptomatic trees), *M. pinitorqua* symptom on the analysed shoot (A – asymptomatic, S – symptomatic), and time point (1 – June 2, 2021, 2 – June 10, 2021, 3 – July 8, 2021). Presented is also the vitality class for each tree at the end of the study (autumn 2021).

| **Disease category** | **Tree ID** | **Vitality** | **Timepoint** | | | | | |
| --- | --- | --- | --- | --- | --- | --- | --- | --- |
|  |  |  | **1** | | **2** | | **3** | |
|  |  |  | **Shoot symptom (*M. pinitorqua*)** | | | | | |
|  |  |  | **A** | **S** | **A** | **S** | **A** | **S** |
| **H** | **2** | **Good** | 1 | 0 | 1 | 0 | 1 | 1 |
|  | **134** | **Good** | 1 | 0 | 1 | 0 | 1 | 1 |
|  | **193** | **Good** | 1 | 0 | 1 | 0 | 1 | 1 |
|  | **248** | **Good** | 1 | 0 | 1 | 0 | 1 | 1 |
|  | **250** | **Good** | 1 | 0 | 1 | 0 | 1 | 1 |
|  | **Total n samples** | | 5 | 0 | 5 | 0 | 5 | 5 |
| **M** | **40** | **Good** | 1 | 1 | 1 | 1 | 1 | 1 |
|  | **44** | **OK** | 1 | 1 | 1 | 1 | 1 | 1 |
|  | **109** | **OK** | 1 | 1 | 1 | 1 | 1 | 1 |
|  | **333** | **OK** | 1 | 1 | 1 | 1 | 1 | 1 |
|  | **390** | **Good** | 1 | 1 | 1 | 1 | 1 | 1 |
|  | **Total n samples** | | 5 | 5 | 5 | 5 | 5 | 5 |
| **MD** | **200** | **Poor** | 1 | 1 | 1 | 1 | 1 | 1 |
|  | **229** | **Poor** | 1 | 1 | 1 | 1 | 1 | 1 |
|  | **258** | **Poor** | 1 | 1 | 1 | 1 | 1 | 1 |
|  | **397** | **Poor** | 1 | 1 | 1 | 1 | 1 | 1 |
|  | **445** | **OK** | 1 | 1 | 1 | 1 | 1 | 1 |
|  | **Total n samples** | | 5 | 5 | 5 | 5 | 5 | 5 |

**TABLE S3** Phenolic compounds analysed by LC-MS/MS [HPLC 1200 (Agilent Technologies)-API3200 (Applied Biosystems)] in negative ionisation mode, Abbreviations: Q1, selected *m/z* of the first quadrupole; Q3, selected *m/z* of the third quadrupole; RF, response factor; DP, declustering potential (V); and CE, collision energy (V). Compound abbreviations are explained in Table 1.

| **Compound** | **Q1** | **Q3** | **RT (min)** | **RF** | **DP** | **CE** |
| --- | --- | --- | --- | --- | --- | --- |
| Apigenin-7-glucoside (internal standard) | 430.8 | 268 | 5.1 |  | -80 | -46 |
| Cat | 288.9 | 109.1 | 4.3 | 8.7 | -30 | -34 |
| Tax | 302.8 | 125.1 | 5.1 | 4.8 | -40 | -28 |
| Ast | 404.8 | 243 | 4.5 | 1.1 | -50 | -38 |
| PAB1 | 576.9 | 289.1 | 4.1 | 5.5 | -50 | -30 |
| Iso | 314.9 | 107.1 | 6.5 | 15.9 | -60 | -46 |
| Pic | 389 | 227 | 4.8 | 0.7 | -50 | -38 |
| QG | 462.9 | 301 | 5.3 | 1.4 | -55 | -40 |
| TaxG | 465 | 285 | 4.7 | 6.7 | -55 | -44 |
| KG | 447 | 284 | 5 | 1.5 | -55 | -30 |
| NCG | 433 | 313 | 4.8 |  | -55 | -30 |
| KAG | 489 | 284 | 5.3 |  | -55 | -30 |
| IAG | 519 | 314 | 5.4 |  | -55 | -30 |
| KCG | 593 | 285 | 5.6 |  | -55 | -30 |
| KDCG | 739 | 285 | 6.8 |  | -55 | -30 |
| MH | 479 | 316 | 4.5 |  | -55 | -30 |
| GCat | 304.9 | 125 | 3.7 |  | -30 | -28 |
| NL | 495 | 167.2 | 4.3 |  | -55 | -36 |
| MTL | 357 | 83 | 6.4 |  | -50 | -38 |

**TABLE S4** Amino acids analysed by LC-MS/MS [HPLC 1260 (Agilent Technologies)-QTRAP6500 (AB SCIEX)] in positive ionisation mode, Abbreviations: Q1, selected *m/z* of the first quadrupole; Q3, selected *m/z* of the third quadrupole; ISQ1, selected *m/z* of the first quadrupole for internal standard; ISQ3, selected *m/z* of the third quadrupole for internal standard; DP, declustering potential (V); and CE, collision energy (V). Amino acid abbreviations are explained in Table 1.

| **Compound** | **Q1** | **Q3** | **RT (min)** |  | **Internal standard** | **IS Q1** | **IS Q3** | **DP** | **CE** |
| --- | --- | --- | --- | --- | --- | --- | --- | --- | --- |
| Ala | 90.1 | 44.1 | 0.5 |  | 13C,15N-Ala | 94.1 | 47.1 | 20 | 17 |
| Ser | 106 | 60.1 | 0.5 |  | 13C,15N-Ser | 110 | 63.1 | 20 | 15 |
| Pro | 116.1 | 70 | 0.5 |  | 13C,15N-Pro | 122.1 | 75 | 20 | 19 |
| Val | 118.1 | 72.2 | 0.5 |  | 13C,15N-Val | 124.1 | 77.2 | 20 | 13 |
| Thr | 120.1 | 74.2 | 0.5 |  | 13C,15N-Thr | 125.1 | 78.2 | 20 | 13 |
| Ile | 132.2 | 86.1 | 1.1 |  | 13C,15N-Ile | 139.2 | 92.1 | 20 | 13 |
| Leu | 132.2 | 86.1 | 1.3 |  | 13C,15N-Leu | 139.2 | 92.1 | 20 | 13 |
| Asp | 134.1 | 74.1 | 0.5 |  | 13C,15N-Asp | 139.1 | 77.1 | 20 | 19 |
| Glu | 148.1 | 102.1 | 0.5 |  | 13C,15N-Glu | 154.1 | 107.1 | 20 | 15 |
| Met | 150.2 | 104.1 | 0.7 |  | 13C,15N-Met | 156.2 | 109.1 | 20 | 13 |
| His | 156.2 | 110.1 | 0.4 |  | 13C,15N-His | 165.2 | 118.1 | 20 | 17 |
| Phe | 166.2 | 120.2 | 2.6 |  | 13C,15N-Phe | 176.2 | 129.2 | 20 | 17 |
| Arg | 175.1 | 70.1 | 0.4 |  | 13C,15N-Arg | 185.1 | 75.1 | 20 | 31 |
| Tyr | 182.1 | 136.2 | 1.4 |  | 13C,15N-Tyr | 192.1 | 145.2 | 20 | 17 |
| Asn | 133.1 | 74.1 | 0.5 |  | 13C,15N-Asp |  |  | 20 | 21 |
| Gln | 147.1 | 130 | 0.5 |  | 13C,15N-Gln | 154.1 | 136 | 20 | 13 |
| Trp | 205.2 | 188.1 | 3.2 |  | D5-Trp | 210 | 193 | 20 | 13 |

**TABLE S5a** Analysed amino acids and their concentrations for all samples and per time point, M. pinitorqua symptom on the analysed shoot (A – asymptomatic, S – symptomatic), disease category (based on the tree’s symptoms in 2020; H – healthy-looking, M – M. pinitorqua-symptomatic, and MD – M. pinitorqua- and D. sapinea-symptomatic), and vitality class in 2021 (nmol/g dw, n = 79). Number of trees are indicated within parentheses. Amino acid abbreviations are explained in Table 1.

|  |  | **Ala** | **Arg** | **Asn** | **Asp** | **GABA** | **Gln** | **Glu** | **His** | **Ile** | **Leu** | **Lys** | **Met** | **Phe** | **Pro** | **Ser** | **Thr** | **Trp** | **Tyr** | **Val** |
| --- | --- | --- | --- | --- | --- | --- | --- | --- | --- | --- | --- | --- | --- | --- | --- | --- | --- | --- | --- | --- |
| **All trees** |  |  |  |  |  |  |  |  |  |  |  |  |  |  |  |  |  |  |  |  |
| (79) | **Mean** | 2711.6 | 107.2 | 361.4 | 966.1 | 19964.4 | 17477.1 | 6994.8 | 757.1 | 381.6 | 394.7 | 152.7 | 40.2 | 324.2 | 872.8 | 3253.5 | 878.3 | 243.2 | 123.0 | 489.3 |
|  | **±SE** | 1466.6 | 180.2 | 290.2 | 482.3 | 14303.7 | 16917.8 | 4035.6 | 335.5 | 154.9 | 177.6 | 60.8 | 21.3 | 130.2 | 1948.2 | 1348.8 | 401.4 | 150.9 | 50.7 | 218.4 |
| **Time point** |  |  |  |  |  |  |  |  |  |  |  |  |  |  |  |  |  |  |  |  |
| **1** | **Mean** | 4341.5 | 158.9 | 541.8 | 1031.9 | 33082.2 | 30280.2 | 8945.8 | 805.9 | 471.1 | 378.1 | 152.5 | 56.5 | 341.3 | 2247.1 | 4406.0 | 1081.8 | 250.9 | 143.5 | 563.6 |
| (24) | **±SE** | 1390.0 | 285.5 | 344.1 | 486.4 | 16756.3 | 23650.0 | 5356.9 | 371.8 | 141.3 | 99.9 | 42.8 | 27.5 | 83.5 | 3158.5 | 1280.2 | 481.3 | 104.6 | 35.9 | 214.6 |
| **2** | **Mean** | 2558.8 | 125.7 | 413.1 | 1061.5 | 18493.4 | 16204.0 | 7754.2 | 1021.8 | 442.0 | 481.2 | 201.8 | 28.5 | 415.5 | 400.3 | 3629.3 | 1015.5 | 370.1 | 153.9 | 613.4 |
| (25) | **±SE** | 589.4 | 140.6 | 276.8 | 333.8 | 8312.6 | 10104.0 | 2458.0 | 231.4 | 146.4 | 252.2 | 63.1 | 8.5 | 150.2 | 160.9 | 752.1 | 325.5 | 173.7 | 51.7 | 215.3 |
| **3** | **Mean** | 1535.1 | 50.5 | 174.1 | 834.1 | 10696.0 | 8295.6 | 4801.2 | 497.5 | 259.6 | 335.9 | 111.9 | 36.9 | 234.5 | 167.3 | 2018.4 | 601.3 | 131.4 | 81.0 | 326.5 |
| (30) | **±SE** | 561.5 | 23.8 | 61.1 | 562.6 | 6106.5 | 4178.8 | 2723.9 | 129.7 | 76.3 | 118.6 | 37.5 | 14.3 | 75.4 | 70.2 | 571.2 | 179.4 | 26.6 | 26.2 | 90.1 |
| **Symptom** |  |  |  |  |  |  |  |  |  |  |  |  |  |  |  |  |  |  |  |  |
| **A** | **Mean** | 2549.1 | 99.9 | 315.5 | 835.5 | 18560.5 | 14974.2 | 6394.0 | 741.2 | 355.1 | 359.7 | 150.9 | 37.9 | 298.4 | 834.0 | 3092.1 | 823.1 | 230.2 | 114.4 | 456.2 |
| (44) | **±SE** | 1485.6 | 210.0 | 228.6 | 389.5 | 13692.4 | 15646.6 | 3779.2 | 338.8 | 125.3 | 100.3 | 56.1 | 19.9 | 91.2 | 2046.4 | 1291.1 | 382.3 | 104.2 | 37.9 | 172.0 |
| **S** | **Mean** | 2916.0 | 116.5 | 419.2 | 1130.4 | 21729.3 | 20623.7 | 7750.2 | 777.1 | 414.8 | 438.8 | 154.9 | 43.2 | 356.7 | 921.7 | 3456.4 | 947.8 | 259.6 | 133.8 | 530.9 |
| (35) | **±SE** | 1437.5 | 136.3 | 347.8 | 540.3 | 15050.0 | 18129.6 | 4271.4 | 335.2 | 182.1 | 236.8 | 67.1 | 22.8 | 162.6 | 1845.7 | 1410.5 | 419.5 | 194.9 | 62.2 | 262.2 |
| **Disease category** |  |  |  |  |  |  |  |  |  |  |  |  |  |  |  |  |  |  |  |  |
| **H** | **Mean** | 2450.2 | 66.5 | 292.9 | 758.5 | 20553.2 | 13249.7 | 5365.7 | 649.7 | 344.5 | 355.8 | 135.6 | 40.3 | 278.9 | 646.6 | 2892.5 | 806.1 | 205.6 | 106.4 | 445.1 |
| (20) | **±SE** | 1395.9 | 28.3 | 157.0 | 460.5 | 16865.2 | 8157.5 | 3095.0 | 291.0 | 121.8 | 100.1 | 62.4 | 22.0 | 84.8 | 1137.8 | 1285.7 | 403.1 | 103.0 | 37.3 | 180.0 |
| **M** | **Mean** | 2814.3 | 107.0 | 388.1 | 1070.8 | 19602.5 | 17902.9 | 7639.3 | 731.5 | 390.8 | 406.4 | 155.7 | 43.4 | 354.4 | 686.3 | 3268.4 | 851.1 | 263.0 | 129.4 | 512.2 |
| (30) | **±SE** | 1361.9 | 133.9 | 291.4 | 472.7 | 13051.2 | 15186.5 | 4634.4 | 253.9 | 160.9 | 229.7 | 65.2 | 24.8 | 163.4 | 750.9 | 1226.5 | 315.9 | 191.5 | 59.6 | 234.7 |
| **MD** | **Mean** | 2785.8 | 135.6 | 381.1 | 1001.0 | 19932.7 | 19952.1 | 7451.6 | 857.8 | 397.6 | 409.5 | 161.4 | 36.9 | 324.3 | 1221.9 | 3487.1 | 956.4 | 248.7 | 127.9 | 496.1 |
| (29) | **±SE** | 1638.0 | 263.4 | 354.2 | 478.6 | 14146.8 | 22225.6 | 3750.5 | 412.3 | 169.2 | 158.1 | 54.4 | 16.3 | 110.8 | 2985.2 | 1496.2 | 473.8 | 129.4 | 47.7 | 227.6 |
| **Vitality** |  |  |  |  |  |  |  |  |  |  |  |  |  |  |  |  |  |  |  |  |
| **Fully vital** | **Mean** | 2438.0 | 67.7 | 290.6 | 810.4 | 18205.6 | 13141.5 | 6276.8 | 658.7 | 339.4 | 348.5 | 141.4 | 39.6 | 282.2 | 576.6 | 2955.5 | 779.1 | 209.7 | 110.2 | 435.2 |
| (32) | **±SE** | 1211.2 | 28.0 | 140.8 | 427.9 | 13982.8 | 7524.4 | 3539.2 | 247.6 | 112.2 | 84.6 | 57.6 | 20.6 | 81.9 | 931.5 | 1195.5 | 334.9 | 92.7 | 38.2 | 158.3 |
| **Mildly affected** | **Mean** | 3060.3 | 116.9 | 409.2 | 1101.6 | 22439.7 | 19168.3 | 7614.8 | 772.2 | 429.3 | 457.3 | 166.6 | 43.4 | 386.8 | 715.9 | 3377.2 | 911.6 | 269.7 | 136.2 | 554.1 |
| (24) | **±SE** | 1466.7 | 147.5 | 316.3 | 473.9 | 13339.6 | 16287.0 | 4864.0 | 316.8 | 161.8 | 254.0 | 68.2 | 25.1 | 167.9 | 792.2 | 1188.7 | 327.3 | 209.6 | 60.4 | 246.1 |
| **Severely affected** | **Mean** | 2728.6 | 152.1 | 410.1 | 1041.5 | 19828.5 | 21744.7 | 7346.8 | 878.4 | 390.4 | 393.7 | 153.9 | 37.8 | 317.4 | 1448.8 | 3539.1 | 981.7 | 262.3 | 127.0 | 497.0 |
| (23) | **±SE** | 1751.7 | 294.7 | 392.3 | 520.4 | 15895.0 | 24660.6 | 3749.6 | 422.1 | 186.5 | 164.3 | 56.4 | 18.0 | 119.7 | 3328.2 | 1651.7 | 524.1 | 140.9 | 53.0 | 248.1 |

**TABLE S5b** Analysed phenolic compounds and their concentrations for all samples and per time point, M. pinitorqua symptom on the analysed shoot (A – asymptomatic, S – symptomatic), disease category (based on the tree’s symptoms in 2020; H – healthy-looking, M – M. pinitorqua-symptomatic, and MD – M. pinitorqua- and D. sapinea-symptomatic), and vitality class in 2021 (n = 80). Compound abbreviations are explained in Table 1.

|  |  | **Ast ^a^** | **Cat ^a^** | **GCat ^b^** | **Iso ^a^** | | **IAG ^b^** | **KDCG ^b^** | **KAG ^b^** | **KCG ^b^** | **KG ^a^** | **MTL ^b^** | **MH ^b^** | **NCG ^b^** | **NL ^b^** | **PAB1 ^a^** | **Pic ^a^** | **QG ^a^** | **Tax ^a^** | **TaxG ^a^** |
| --- | --- | --- | --- | --- | --- | --- | --- | --- | --- | --- | --- | --- | --- | --- | --- | --- | --- | --- | --- | --- |
| **All trees** |  |  |  |  |  | |  |  |  |  |  |  |  |  |  |  |  |  |  |  |
| (80) | **Mean** | 1.5 | 1580 | 21 | 12.5 | | 24 | 18 | 24 | 13 | 598 | 2 | 17 | 6 | 16 | 323 | 0.3 | 124 | 306 | 1025 |
|  | **±SE** | 1.5 | 867 | 9 | 16.7 | | 16 | 13 | 18 | 9 | 441 | 4 | 17 | 9 | 8 | 271 | 0.3 | 103 | 388 | 1009 |
| **Time point** | |  |  |  |  | |  |  |  |  |  |  |  |  |  |  |  |  |  |  |
| **1** | **Mean** | 1.6 | 1526 | 30 | 2.6 | | 30 | 11 | 33 | 15 | 952 | 4 | 26 | 12 | 9 | 252 | 0.3 | 188 | 54 | 284 |
| (25) | **±SE** | 1.2 | 378 | 7 | 1.3 | | 12 | 5 | 15 | 8 | 278 | 6 | 15 | 12 | 3 | 127 | 0.1 | 72 | 23 | 204 |
| **2** | **Mean** | 0.9 | 1084 | 23 | 8.1 | | 32 | 34 | 30 | 19 | 765 | 1 | 26 | 6 | 15 | 188 | 0.2 | 173 | 52 | 435 |
| (25) | **±SE** | 0.7 | 307 | 6 | 14.4 | | 18 | 10 | 20 | 9 | 364 | 4 | 17 | 7 | 5 | 115 | 0.1 | 103 | 25 | 495 |
| **3** | **Mean** | 1.9 | 2038 | 13 | 24.5 | | 13 | 11 | 11 | 6 | 162 | 0 | 3 | 0 | 22 | 495 | 0.4 | 29 | 728 | 2134 |
| (30) | **±SE** | 2.0 | 1191 | 4 | 18.2 | | 9 | 6 | 11 | 4 | 165 | 1 | 4 | 0 | 7 | 354 | 0.4 | 24 | 338 | 699 |
| **Symptom** | |  |  |  |  | |  |  |  |  |  |  |  |  |  |  |  |  |  |  |
| **A** | **Mean** | 1.1 | 1243 | 22 | 11.4 | | 26 | 20 | 27 | 14 | 684 | 1 | 21 | 7 | 16 | 200 | 0.2 | 142 | 245 | 954 |
| (45) | **±SE** | 0.9 | 326 | 9 | 16.4 | | 16 | 15 | 18 | 10 | 447 | 2 | 19 | 9 | 8 | 87 | 0.1 | 109 | 337 | 889 |
| **S** | **Mean** | 2.0 | 2012 | 20 | 14.0 | | 21 | 15 | 20 | 11 | 487 | 3 | 13 | 5 | 15 | 481 | 0.4 | 101 | 385 | 1116 |
| (35) | **±SE** | 1.9 | 1125 | 9 | 17.1 | | 16 | 10 | 17 | 8 | 413 | 5 | 12 | 8 | 7 | 339 | 0.4 | 91 | 437 | 1151 |
| **Disease category** | |  |  |  |  | |  |  |  |  |  |  |  |  |  |  |  |  |  |  |
| **H** | **Mean** | 1.2 | 1477 | 17 | 8.7 | | 15 | 14 | 14 | 8 | 405 | 0 | 8 | 4 | 15 | 293 | 0.2 | 85 | 289 | 1201 |
| (20) | **±SE** | 0.9 | 657 | 6 | 10.0 | | 11 | 12 | 13 | 7 | 380 | 1 | 10 | 6 | 7 | 224 | 0.2 | 86 | 280 | 878 |
| **M** | **Mean** | 1.6 | 1465 | 24 | 14.0 | | 24 | 18 | 24 | 16 | 611 | 1 | 19 | 8 | 15 | 299 | 0.3 | 115 | 335 | 864 |
| (30) | **±SE** | 1.3 | 832 | 10 | 17.9 | | 12 | 13 | 14 | 9 | 396 | 2 | 17 | 10 | 9 | 301 | 0.2 | 74 | 461 | 1037 |
| **MD** | **Mean** | 1.5 | 1762 | 22 | 13.7 | | 30 | 21 | 30 | 14 | 713 | 3 | 22 | 5 | 17 | 368 | 0.3 | 159 | 289 | 1069 |
| (30) | **±SE** | 1.9 | 1008 | 9 | 18.9 | | 19 | 14 | 22 | 9 | 488 | 6 | 19 | 9 | 7 | 271 | 0.4 | 127 | 380 | 1069 |
| **Vitality** |  |  |  |  |  | |  |  |  |  |  |  |  |  |  |  |  |  |  |  |
| **Fully vital** | **Mean** | 1.4 | 1448 | 20 | 9.7 | | 19 | 16 | 18 | 11 | 483 | 0 | 12 | 3 | 15 | 285 | 0.3 | 95 | 358 | 1169 |
| (32) | **±SE** | 1.2 | 660 | 8 | 12.0 | | 13 | 12 | 13 | 9 | 390 | 1 | 14 | 6 | 7 | 227 | 0.2 | 82 | 444 | 1037 |
| **Mildly affected** | **Mean** | 1.3 | 1634 | 24 | 14.2 | | 24 | 18 | 24 | 14 | 643 | 2 | 21 | 12 | 15 | 332 | 0.3 | 134 | 232 | 754 |
| (24) | **±SE** | 1.2 | 910 | 11 | 17.7 | | 15 | 14 | 17 | 10 | 453 | 4 | 18 | 12 | 8 | 315 | 0.2 | 101 | 274 | 773 |
| **Severely affected** | **Mean** | 1.8 | 1701 | 22 | 14.7 | | 31 | 22 | 31 | 14 | 705 | 3 | 21 | 3 | 18 | 366 | 0.3 | 153 | 312 | 1106 |
| (24) | **±SE** | 2.0 | 1059 | 8 | 20.7 | | 18 | 14 | 22 | 8 | 475 | 6 | 19 | 6 | 7 | 283 | 0.4 | 121 | 408 | 1155 |
| ^a^ Absolute quantity (µg/g dw)  ^b^ Relative quantity(normalised peak area/g dw) | | | | | |  |  |  |  |  |  |  |  |  |  |  |  |  |  |  |

**
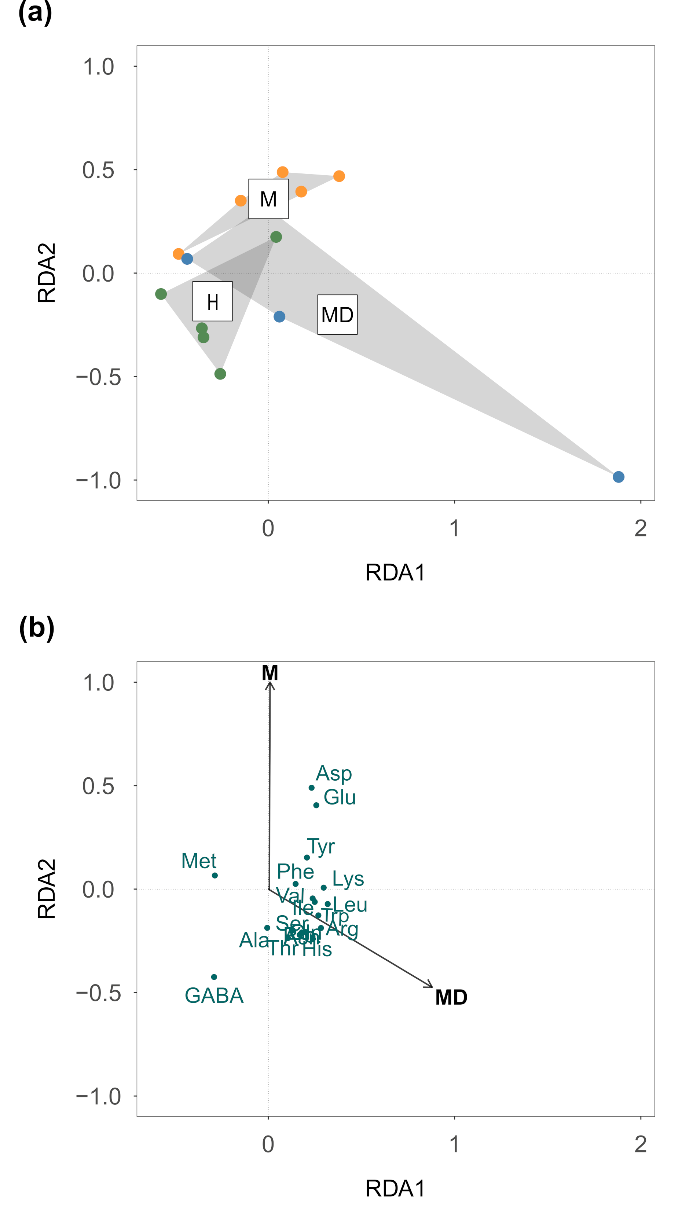

FIGURE S4** Composition of amino acids in asymptomatic shoots at time point 1 (June 2, 2021) explained by disease category based on the tree’s symptoms in 2020; H – healthy-looking, M – *M. pinitorqua*-symptomatic, and MD – *M. pinitorqua*- and *D. sapinea*-symptomatic. a) Sample plot presented with hulls connecting the samples from each disease category. b) The RDA presented with loadings. The disease category was not associated with the composition of amino acids (RDA; permutational ANOVA; p = 0.404, adj. R^2^ = 0.009). For full compound names please refer to Table 1.

**
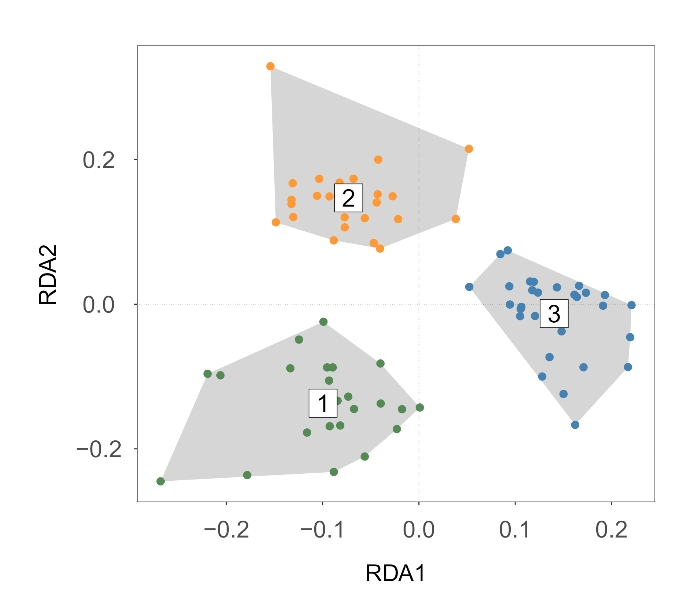

FIGURE S5** Metabolite profiles explained by sampling time point ; 1 – June 2, 2021, 2 – June 10, 2021, 3 – July 8, 2021. The composition of metabolites was linked to the time point (RDA conditioned on *M. pinitorqua* symptom on the analysed shoot and tree individual. Permutational ANOVA; p = 0.001 ***, adj. R^2^ = 0.418).

**TABLE S6a** Results of redundancy analysis (RDA) on phenolic compounds and amino acids in asymptomatic shoots at time point 1, explained by disease category.

| **Results of redundancy analysis (RDA) on phenolic compounds in asymptomatic shoots** | | | | | |
| --- | --- | --- | --- | --- | --- |
| **at time point 1, explained by disease category.** | | |  |  |  |
|  |  |  |  |  |  |
| **Permutational ANOVA on model** | |  |  |  |  |
|  | Df | Variance | F | p |  |
| Disease category | 2 | 5.9255 | 2.9445 | 0.001 | *** |
| Residual | 12 | 12.0745 |  |  |  |
|  |  |  |  |  |  |
|  | R^2^ = 0.329 | Adj. R^2^ = 0.217 |  |  |  |
|  |  |  |  |  |  |
| **Partitioning of correlations** | |  |  |  |  |
|  | Inertia | Proportion |  |  |  |
| Total | 18.00 | 1.00 |  |  |  |
| Constrained | 5.926 | 0.3292 |  |  |  |
| Unconstrained | 12.074 | 0.6708 |  |  |  |
|  |  |  |  |  |  |
| **Importance of components** | |  |  |  |  |
|  | RDA1 | RDA2 | PC1 |  |  |
| Eigenvalue | 4.5889 | 1.33666 | 3.5759 |  |  |
| Proportion explained | 0.2549 | 0.07426 | 0.1987 |  |  |
| Cumulative proportion | 0.2549 | 0.3292 | 0.5279 |  |  |
| Test of significance | p = 0.001 | p = 0.195 |  |  |  |
|  |  |  |  |  |  |
| **Biplot scores for constraining variables** | | |  |  |  |
|  | RDA1 | RDA2 |  |  |  |
| Disease category M | -0.1869 | 0.9824 |  |  |  |
| Disease category MD | -0.7573 | -0.6531 |  |  |  |
|  |  |  |  |  |  |
| **Results of redundancy analysis (RDA) on amino acids in asymptomatic shoots** | | | | |  |
| **at time point 1, explained by disease category.** | | |  |  |  |
|  |  |  |  |  |  |
| **Permutational ANOVA on model** | |  |  |  |  |
|  | Df | Variance | F | p |  |
| Disease category | 2 | 3.0636 | 1.0573 | 0.378 |  |
| Residual | 11 | 15.9364 |  |  |  |
|  |  |  |  |  |  |
|  | R^2^ = 0.161 | Adj. R^2^ = 0.009 |  |  |  |
|  |  |  |  |  |  |
| **Partitioning of correlations** | |  |  |  |  |
|  | Inertia | Proportion |  |  |  |
| Total | 19 | 1 |  |  |  |
| Constrained | 3.064 | 0.1612 |  |  |  |
| Unconstrained | 15.936 | 0.8388 |  |  |  |
|  |  |  |  |  |  |
| **Importance of components** | |  |  |  |  |
|  | RDA1 | RDA2 | PC1 |  |  |
| Eigenvalue | 1.77335 | 1.29029 | 10.9863 |  |  |
| Proportion explained | 0.09333 | 0.06791 | 0.5782 |  |  |
| Cumulative proportion | 0.09333 | 0.16124 | 0.7395 |  |  |
| Test of significance | p = 0.675 | p = 0.469 |  |  |  |
|  |  |  |  |  |  |
| **Biplot scores for constraining variables** | | |  |  |  |
|  | RDA1 | RDA2 |  |  |  |
| Disease category M | 0.0038 | 1 |  |  |  |
| Disease category MD | 0.8801 | -0.4748 |  |  |  |
|  |  |  |  |  |  |

**TABLE S6b**

| **Kruskal-Wallis test results assessing the relationship between total phenolics and disease category in asymptomatic shoots at time point 1.** | | | | | |
| --- | --- | --- | --- | --- | --- |
|  |  |  |  |  |  |
| Kruskal-Wallis chi-squared = 7.46, df = 2, p-value = 0.02399 | | | | |  |
|  |  |  |  |  |  |
| **Dunn's test results comparing total phenolics across disease categories.** | | | | | |
|  |  |  |  |  |  |
| Comparisons | H - M | H - MD | M - MD |  |  |
| z score | -0.9192388 | -2.68701 | -1.76777 |  |  |
| p value | 0.178985336 | 0.003605 | 0.03855 |  |  |
|  |  |  |  |  |  |
| **Kruskal-Wallis test results assessing the relationship between total amino acids and disease category in asymptomatic shoots at time point 1.** | | | | | |
|  |  |  |  |  |  |
| Kruskal-Wallis chi-squared = 2.7165, df = 2, p-value = 0.2571 | | | | |  |
|  |  |  |  |  |  |

**TABLE S6c**

| **Results of ANOVA on linear model testing for differences in *M. pinitorqua* DNA copies between different time points and shoot symptom, and the interaction between time point and shoot symptom.** | | | | | | | | | | | | | | | | | |  |  |
| --- | --- | --- | --- | --- | --- | --- | --- | --- | --- | --- | --- | --- | --- | --- | --- | --- | --- | --- | --- |
|  |  |  |  |  |  | |  | |  | |  | |  | | |  | | |  |
|  |  |  | Sum of Squares | Mean Square |  | |  | |  | |  | |  | | |  | | |  |
|  |  | Df |  |  | F value | | p value | |  | |  | |  | | |  | | |  |
| Time point |  | 2 | 121.67 | 60.83 | 13.161 | | 1.35E-05 | | *** | |  | |  | | |  | | |  |
| Shoot symptom |  | 1 | 417.84 | 417.84 | 90.398 | | 2.39E-14 | | *** | |  | |  | | |  | | |  |
| Time point:Shoot symptom | | 2 | 72.86 | 36.43 | 7.881 | | 0.000803 | | *** | |  | |  | | |  | | |  |
| Residuals |  | 72 | 332.8 | 4.62 |  | |  | |  | |  | |  | | |  | | |  |
|  |  |  |  |  |  | |  | |  | |  | |  | | |  | | |  |
| **Results of estimated marginal means testing for differences in *M. pinitorqua* DNA copies between different shoot symptoms** | | | | | | | | | | | | | | | | | |  |  |
| **per time point (adjusted for multivariate t distribution, 95% confidence level).** | | | | | | | | | |  | |  | |  | | |  | | |
|  |  |  |  |  |  | |  | |  | |  | |  | | |  | | |  |
| Contrast |  | Symptom | Estimate | SE | df | | Lower CL | | Upper CL | | t ratio | | p value | | |  | | |  |
| Time point 1 - Time point 2 | | A | 1.35 | 0.785 | 72 | | -0.524 | | 3.23 | | 1.73 | | 0.203 | | |  | | |  |
| Time point 1 - Time point 3 | | A | 2.44 | 0.815 | 72 | | 0.492 | | 4.39 | | 3 | | 0.0103 | | | * | | |  |
| Time point 2 - Time point 3 | | A | 1.09 | 0.815 | 72 | | -0.863 | | 3.04 | | 1.33 | | 0.381 | | |  | | |  |
| Time point 1 - Time point 2 | | S | -1.92 | 0.961 | 72 | | -4.22 | | 0.381 | | -2 | | 0.12 | | |  | | |  |
| Time point 1 - Time point 3 | | S | 3.84 | 0.878 | 72 | | 1.74 | | 5.94 | | 4.38 | | 0.000117 | | | *** | | |  |
| Time point 2 - Time point 3 | | S | 5.76 | 0.878 | 72 | | 3.66 | | 7.86 | | 6.57 | | 2.06E-08 | | | *** | | |  |
|  |  |  |  |  |  | |  | |  | |  | |  | | |  | | |  |
| **Results of estimated marginal means testing for differences in *M. pinitorqua* DNA copies between different time points** | | | | | | | | | | | | | | |  | | |  |  |
| **(adjusted for multivariate t distribution, 95% confidence level).** | | | | | |  | |  | |  | |  | |  | | |  | | |
|  |  |  |  |  |  | |  | |  | |  | |  | | |  | | |  |
| Contrast |  | Estimate | SE | df | Lower CL | | Upper CL | | t ratio | | p value | |  | | |  | | |  |
| Time point 1 - Time point 2 | | -0.283 | 0.621 | 72 | -1.77 | | 1.2 | | -0.456 | | 0.892 | |  | | |  | | |  |
| Time point 1 - Time point 3 | | 3.14 | 0.599 | 72 | 1.71 | | 4.57 | | 5.25 | | 3.34E-06 | | *** | | |  | | |  |
| Time point 2 - Time point 3 | | 3.42 | 0.599 | 72 | 1.99 | | 4.86 | | 5.72 | | 6.41E-07 | | *** | | |  | | |  |
|  |  |  |  |  |  | |  | |  | |  | |  | | |  | | |  |
| **Results of estimated marginal means testing for differences in *M. pinitorqua* DNA copies between different shoot symptoms** | | | | | | | | | | | | | | | | | |  |  |
| **(adjusted for multivariate t distribution, 95% confidence level).** | | | | | |  | |  | |  | |  | |  | | |  | | |
|  |  |  |  |  |  | |  | |  | |  | |  | | |  | | |  |
| Contrast |  | Estimate | SE | df | Lower CL | | Upper CL | | t ratio | | p value | |  | | |  | | |  |
| Asymptomatic - Symptomatic | | -4.8 | 0.495 | 72 | -5.78 | | -3.81 | | -9.69 | | 1.09E-14 | | *** | | |  | | |  |
|  |  |  |  |  |  | |  | |  | |  | |  | | |  | | |  |
| **Results of ANOVA on linear model testing for differences in *M. pinitorqua* DNA copies between different disease categories.** | | | | | | | | | | | | | | | | | |  |  |
|  |  |  |  |  |  | |  | |  | |  | |  | | |  | | |  |
|  |  | Sum of Squares | Mean Square |  |  | |  | |  | |  | |  | | |  | | |  |
|  | Df |  |  | F value | p value | |  | |  | |  | |  | | |  | | |  |
| Disease category | 2 | 223.14 | 111.568 | 11.589 | 4.11E-05 | | *** | |  | |  | |  | | |  | | |  |
| Residuals | 75 | 722.03 | 9.627 |  |  | |  | |  | |  | |  | | |  | | |  |
|  |  |  |  |  |  | |  | |  | |  | |  | | |  | | |  |
| **Results of estimated marginal means testing for differences in *M. pinitorqua* DNA copies between different disease categories (adjusted for multivariate t distribution, 95% confidence level).** | | | | | | | | | | | | | | | | | |  |  |
|  |  |  |  |  |  | |  | |  | |  | |  | | |  | | |  |
| Contrast | Estimate | SE | df | Lower CL | Upper CL | | t ratio | | p value | |  | |  | | |  | | |  |
| H - M | -2.64 | 0.902 | 75 | -4.79 | -0.483 | | -2.93 | | 0.0125 | | * | |  | | |  | | |  |
| H - MD | -4.34 | 0.902 | 75 | -6.5 | -2.19 | | -4.81 | | 2.35E-05 | | *** | |  | | |  | | |  |
| M - MD | -1.7 | 0.815 | 75 | -3.65 | 0.245 | | -2.09 | | 0.0986 | | . | |  | | |  | | |  |
|  |  |  |  |  |  | |  | |  | |  | |  | | |  | | |  |

**TABLE S6d**

| **Results of ANOVA on linear model testing for differences in *D. sapinea* DNA copies between different time points and shoot symptom, and the interaction between time point and shoot symptom.** | | | | | | | | | |
| --- | --- | --- | --- | --- | --- | --- | --- | --- | --- |
|  |  |  |  |  |  |  |  |  |  |
|  |  |  | Sum of Squares | Mean Square |  |  |  |  |  |
|  |  | Df |  |  | F value | p value |  |  |  |
| Time point |  | 2 | 185.885 | 92.943 | 130.1027 | <2e-16 | *** |  |  |
| Shoot symptom |  | 1 | 0.066 | 0.066 | 0.0928 | 0.7615 |  |  |  |
| Time point:Shoot symptom | | 2 | 0.107 | 0.053 | 0.0748 | 0.928 |  |  |  |
| Residuals |  | 74 | 52.864 | 0.714 |  |  |  |  |  |
|  |  |  |  |  |  |  |  |  |  |
| **Results of estimated marginal means testing for differences in *D. sapinea* DNA copies between different time points (adjusted for multivariate t distribution, 95% confidence level).** | | | | | | | | | |
|  |  |  |  |  |  |  |  |  |  |
| Contrast |  | Estimate | SE | df | Lower CL | Upper CL | t ratio | p value |  |
| Time point 1 - Time point 2 | | -0.886 | 0.235 | 77 | -1.45 | -0.325 | -3.77 | 0.00091 | *** |
| Time point 1 - Time point 3 | | -3.51 | 0.225 | 77 | -4.04 | -2.97 | -15.6 | 0 | *** |
| Time point 2 - Time point 3 | | -2.62 | 0.225 | 77 | -3.16 | -2.08 | -11.7 | 0 | *** |
|  |  |  |  |  |  |  |  |  |  |
| **Results of ANOVA on linear model testing for differences in *D. sapinea* DNA copies between different disease categories.** | | | | | | | | | |
|  | |  |  |  |  |  |  |  |  |
|  |  | Sum of Squares | Mean Square |  |  |  |  |  |  |
|  | Df |  |  | F value | p value |  |  |  |  |
| Disease category | 2 | 0.702 | 0.35122 | 0.1135 | 0.8928 |  |  |  |  |
| Residuals | 77 | 238.22 | 3.09377 |  |  |  |  |  |  |
|  |  |  |  |  |  |  |  |  |  |

**TABLE S6e**

| **Results of redundancy analysis (RDA) on amino acids and phenolic compounds explained** | | | | | | | |
| --- | --- | --- | --- | --- | --- | --- | --- |
| **by time point, and conditioned on shoot symptom and tree individual.** | | | | | |  |  |
|  |  |  |  |  |  |  |  |
| **Permutational ANOVA on model** | |  |  |  |  |  |  |
|  | Df | Variance | F | p |  |  |  |
| Time point | 2 | 13.013 | 25.508 | 0.001 | *** |  |  |
| Residual | 61 | 15.56 |  |  |  |  |  |
|  |  |  |  |  |  |  |  |
|  | R^2^ = 0.352 | Adj. R^2^ = 0.418 |  |  |  |  |  |
|  |  |  |  |  |  |  |  |
| **Partitioning of correlations** | |  |  |  |  |  |  |
|  | Inertia | Proportion |  |  |  |  |  |
| Total | 37.00 | 1.00 |  |  |  |  |  |
| Conditioned | 8.427 | 0.2278 |  |  |  |  |  |
| Constrained | 13.013 | 0.3517 |  |  |  |  |  |
| Unconstrained | 15.56 | 0.4205 |  |  |  |  |  |
|  |  |  |  |  |  |  |  |
| **Importance of components** | |  |  |  |  |  |  |
|  | RDA1 | RDA2 | PC1 |  |  |  |  |
| Eigenvalue | 10.6346 | 2.37856 | 4.9011 |  |  |  |  |
| Proportion explained | 0.3722 | 0.08324 | 0.1715 |  |  |  |  |
| Cumulative proportion | 0.3722 | 0.45543 | 0.627 |  |  |  |  |
| Test of significance | p = 0.001 | p = 0.001 |  |  |  |  |  |
|  |  |  |  |  |  |  |  |
| **Biplot scores for constraining variables** | | |  |  |  |  |  |
|  | RDA1 | RDA2 |  |  |  |  |  |
| Time point 2 | -0.4415 | 0.889 |  |  |  |  |  |
| Time point 3 | 0.977 | -0.09006 |  |  |  |  |  |
|  |  |  |  |  |  |  |  |

**TABLE S6f**

| **Results of redundancy analysis (RDA) on amino acids explained by shoot symptom,** | | | | |  |
| --- | --- | --- | --- | --- | --- |
| **and conditioned on time point and tree individual.** | | |  |  |  |
|  |  |  |  |  |  |
| **Permutational ANOVA on model** | |  |  |  |  |
|  | Df | Variance | F | p |  |
| Shoot symptom | 1 | 0.6468 | 4.4155 | 0.006 | ** |
| Residual | 61 | 8.9359 |  |  |  |
|  |  |  |  |  |  |
|  | R^2^ = 0.034 | Adj. R^2^ = 0.033 |  |  |  |
|  |  |  |  |  |  |
| **Partitioning of correlations** | |  |  |  |  |
|  | Inertia | Proportion |  |  |  |
| Total | 19.00 | 1.00 |  |  |  |
| Conditioned | 9.4173 | 0.49565 |  |  |  |
| Constrained | 0.6468 | 0.03404 |  |  |  |
| Unconstrained | 8.9359 | 0.47031 |  |  |  |
|  |  |  |  |  |  |
| **Importance of components** | |  |  |  |  |
|  | RDA1 | PC1 | PC2 |  |  |
| Eigenvalue | 0.6468 | 4.567 | 1.4108 |  |  |
| Proportion explained | 0.0675 | 0.4766 | 0.1472 |  |  |
| Cumulative proportion | 0.0675 | 0.5441 | 0.6913 |  |  |
| Test of significance | p = 0.013 |  |  |  |  |
|  |  |  |  |  |  |
| **Biplot scores for constraining variables** | | |  |  |  |
|  | RDA1 |  |  |  |  |
| Symptomatic shoot | 0.965 |  |  |  |  |
|  |  |  |  |  |  |

**TABLE S6g**

| **Results of redundancy analysis (RDA) on phenolic compounds explained by shoot symptom, and conditioned on time point and tree individual.** | | | | | |
| --- | --- | --- | --- | --- | --- |
|  |  |  |  |  |  |
| **Permutational ANOVA on model** | |  |  |  |  |
|  | Df | Variance | F | p |  |
| Shoot symptom | 1 | 0.9243 | 8.7196 | 0.001 | *** |
| Residual | 62 | 6.5721 |  |  |  |
|  |  |  |  |  |  |
|  | R^2^ = 0.051 | Adj. R^2^ = 0.057 |  |  |  |
|  |  |  |  |  |  |
| **Partitioning of correlations** | |  |  |  |  |
|  | Inertia | Proportion |  |  |  |
| Total | 18.00 | 1.00 |  |  |  |
| Conditioned | 10.5037 | 0.58354 |  |  |  |
| Constrained | 0.9243 | 0.05135 |  |  |  |
| Unconstrained | 6.5721 | 0.36511 |  |  |  |
|  |  |  |  |  |  |
| **Importance of components** | |  |  |  |  |
|  | RDA1 | PC1 | PC2 |  |  |
| Eigenvalue | 0.9243 | 2.26 | 1.5464 |  |  |
| Proportion explained | 0.1233 | 0.3015 | 0.2063 |  |  |
| Cumulative proportion | 0.1233 | 0.4248 | 0.6311 |  |  |
| Test of significance | p = 0.001 |  |  |  |  |
|  |  |  |  |  |  |
| **Biplot scores for constraining variables** | | |  |  |  |
|  | RDA1 |  |  |  |  |
| Symptomatic shoot | 0.967 |  |  |  |  |
|  |  |  |  |  |  |

**TABLE S6h**

| **Wilcoxon rank sum exact test results for evaluating the relationship between total phenolics and shoot symptom at each time point.** | | | |
| --- | --- | --- | --- |
|  |  |  |  |
| Time point | W | p value |  |
| 1 | 72 | 0.8918 |  |
| 2 | 108 | 0.07092 |  |
| 3 | 32 | 0.0004781 |  |
|  |  |  |  |
| **Wilcoxon rank sum exact test results for evaluating the relationship between total amino acids and shoot symptom at each time point.** | | | |
|  |  |  |  |
| Time point | W | p value |  |
| 1 | 50 | 0.2591 |  |
| 2 | 56 | 0.311 |  |
| 3 | 31 | 0.000394 |  |
|  |  |  |  |

**TABLE S6i**

| **Results of redundancy analysis (RDA) on phenolic compounds explained by the interaction between shoot symptom and abundance of D. sapinea DNA, and conditioned on time point and tree individual.** | | | | | | |
| --- | --- | --- | --- | --- | --- | --- |
|  |  |  |  |  |  |  |
| **Permutational ANOVA on model** | |  |  |  |  |  |
|  | Df | Variance | F | p |  |  |
| Shoot symptom | 1 | 0.9243 | 9.6722 | 0.001 | *** |  |
| *D. sapinea* DNA | 1 | 0.1077 | 1.1265 | 0.331 |  |  |
| Symptom × *D.s* DNA | 1 | 0.7307 | 7.6466 | 0.001 | *** |  |
| Residual | 60 | 5.7337 |  |  |  |  |
|  |  |  |  |  |  |  |
|  | R^2^ = 0.100 | Adj. R^2^ = 0.106 |  |  |  |  |
|  |  |  |  |  |  |  |
| **Partitioning of correlations** | |  |  |  |  |  |
|  | Inertia | Proportion |  |  |  |  |
| Total | 18.00 | 1.00 |  |  |  |  |
| Conditioned | 10.504 | 0.5835 |  |  |  |  |
| Constrained | 1.809 | 0.1005 |  |  |  |  |
| Unconstrained | 5.687 | 0.316 |  |  |  |  |
|  |  |  |  |  |  |  |
| **Importance of components** | |  |  |  |  |  |
|  | RDA1 | RDA2 | RDA3 |  |  |  |
| Eigenvalue | 1.4562 | 0.3058 | 0.046913 |  |  |  |
| Proportion explained | 0.1943 | 0.0408 | 0.006258 |  |  |  |
| Cumulative proportion | 0.1943 | 0.2351 | 0.241317 |  |  |  |
| Test of significance | p = 0.001 | p = 0.036 | p = 0.832 |  |  |  |
|  |  |  |  |  |  |  |
| **Biplot scores for constraining variables** | | |  |  |  |  |
|  | RDA1 | RDA2 | RDA3 |  |  |  |
| Symptomatic shoot | 0.71868 | -0.5976 | 0.2478 |  |  |  |
| *D. sapinea* DNA | 0.04486 | 0.2478 | 0.4601 |  |  |  |
| S shoot × *D.s* DNA | 0.75473 | 0.269 | 0.2058 |  |  |  |
|  |  |  |  |  |  |  |
